# Supplementary material for: Biopsychosocial Intervention for Stroke Carers (BISC): protocol for a feasibility randomised controlled trial (RCT)
Source: BMJ Open. 2017 Oct 22;7(10):e018309. doi: 10.1136/bmjopen-2017-018309 (PMC5665302; doi:10.1136/bmjopen-2017-018309)
Supplement: Author's manuscript [file bmjopen-2017-018309.draft_revisions.pdf]

BMJ Open is committed to open peer review. As part of this commitment we make the peer review history of every article we publish publicly available.

When an article is published we post the peer reviewers' comments and the authors' responses online. We also post the versions of the paper that were used during peer review. These are the versions that the peer review comments apply to.

The versions of the paper that follow are the versions that were submitted during the peer review process. They are not the versions of record or the final published versions. They should not be cited or distributed as the published version of this manuscript.

BMJ Open is an open access journal and the full, final, typeset and author-corrected version of record of the manuscript is available on our site with no access controls, subscription charges or pay-per-view fees (http://bmjopen.bmj.com).

If you have any questions on BMJ Open's open peer review process please email editorial.bmjopen@bmj.com

BMJ Open

Biopsychosocial Intervention for Stroke Carers (BISC): Protocol for a Feasibility Randomised Controlled Trial (RCT)

Journal:	*BMJ Open*
Manuscript ID	bmjopen-2017-018309
Article Type:	Protocol
Date Submitted by the Author:	21-Jun-2017
Complete List of Authors:	Walker, Marion; Faculty of Medicine & Health Sciences, University of Nottingham<br>Thomas, Shirley<br>Whitehead, Phillip; University of Nottingham, Division of Rehabilitation and Ageing<br>Condon, Laura<br>Fisher, Rebecca; Univ Nottingham<br>Cobley, Christine; Derbyshire Healthcare NHS Foundation Trust
**Primary Subject Heading**:	Health services research
Secondary Subject Heading:	Rehabilitation medicine, Neurology
Keywords:	Carers, Stroke < NEUROLOGY, Biospsychosocial model

SCHOLARONE™
Manuscripts

w only

Biopsychosocial Intervention for Stroke Carers (BISC): Protocol for a Feasibility Randomised Controlled Trial (RCT)

Marion F Walker*
Division of Rehabilitation and Ageing, University of Nottingham
B Floor, The Medical School, Queens Medical Centre, Nottingham, NG7 2UH
marion.walker@nottingham.ac.uk

Shirley A Thomas
Division of Rehabilitation and Ageing, University of Nottingham
B Floor, The Medical School, Queens Medical Centre, Nottingham, NG7 2UH
shirley.thomas@nottingham.ac.uk

Phillip J Whitehead
Division of Rehabilitation and Ageing, University of Nottingham
B Floor, The Medical School, Queens Medical Centre, Nottingham, NG7 2UH
phillip.j.whitehead@nottingham.ac.uk

Laura Condon
Division of Rehabilitation and Ageing, University of Nottingham
B Floor, The Medical School, Queens Medical Centre, Nottingham, NG7 2UH
laura.condon@nottingham.ac.uk

Rebecca Fisher
Division of Rehabilitation and Ageing, University of Nottingham
B Floor, The Medical School, Queens Medical Centre, Nottingham, NG7 2UH
rebecca.fisher@nottingham.ac.uk

Christine Cobley
Department of Clinical Psychology, Derbyshire Healthcare NHS Foundation Trust, St Andrews House, 201 London Road, Derby, DE1 2TZ.
Christine.cobley@derbyshcft.nhs.uk

*Corresponding Author

Word Count (main text): 4062

Protocol Version 5.0 17.10.16

For peer review only - http://bmjopen.bmj.com/site/about/guidelines.xhtml

Abstract

Introduction

Reducing length of hospital stay for stroke survivors often creates a rapid shift in the responsibility of care towards informal carers. Adjustment to the caregiving process is experienced by many carers as overwhelming, complex and demanding, and can have a detrimental impact on mental and physical health and wellbeing. National policy guidelines recommend that carers' needs are considered and addressed; despite this, few interventions have been developed and empirically evaluated. We developed a biopsychosocial intervention in collaboration with carers of stroke survivors. Our aim is to determine whether the intervention can be delivered in a group setting and evaluated using a randomised controlled trial (RCT).

Methods and Analysis

Feasibility randomised controlled trial (RCT) and nested qualitative interview study. We aim to recruit up to 40 dyads of stroke survivors and carers within one year of the stroke onset. Dyads are randomised to usual care or usual care plus biopsychosocial intervention. Each intervention group will consist of five stroke carers. The intervention will focus on: psychoeducation, psychological adjustment to stroke, strategies for reducing unwanted negative thoughts and emotions, and problem solving strategies. The main outcome is the feasibility of conducting an RCT. Participant outcomes include: anxiety and depression, quality of life, and carer strain.

Ethics and Dissemination

Favourable ethical opinion was provided by East Midlands – Nottingham 2 Research Ethics Committee (14/EMI/1264). This study will determine whether delivery of the biopsychosocial intervention is feasible and acceptable to stroke carers within a group format. It will also determine whether it is feasible to evaluate the effects of the biopsychosocial intervention in an RCT. We will disseminate our findings through peer-reviewed publications and presentations at national and international conferences.

Trial Registration: ISRCTN 15643456

For peer review only

For peer review only - http://bmjopen.bmj.com/site/about/guidelines.xhtml

Strengths and limitations of this study

- Enhancing the wellbeing of carers is a national priority. However, few interventions for carers have been developed or evaluated.
- This is a pragmatic trial conducted in a real world setting. The intervention content is based on the findings from developmental work with carers of stroke survivors and stroke rehabilitation experts.
- This feasibility study will be conducted in a single site only.
- The intervention focusses only on the initial stages of carer support (up to one year post-stroke onset). Significant problems may develop for carers at later stages which need to be identified and referred for more intensive/specialist support.

For peer review only

INTRODUCTION

Background and Rationale

A carer has been defined as "a person of any age who provides unpaid help and support to a relative, friend or neighbour who cannot manage to live independently without the carer's help due to frailty, illness, disability or addiction"[1]. Carers play a vital role in the early rehabilitation process and long-term management of the stroke survivor[2]. Carers deal with a range of care needs and demands including mobility, self-care, communication difficulties as well as cognitive impairment, mood and personality changes in the stroke survivor[3].

Policy initiatives emphasise the need for shorter inpatient hospital stays which has increased the reliance on informal care within the community for stroke survivors[4]. Thus, a growing number of people are unexpectedly finding themselves in the caregiving role. The increased provision of informal care places carers at elevated risk of poorer mental and physical health, accompanied by reduced opportunity for paid employment and social interaction and activity[3]. An estimate of the psychosocial impact that might be associated with stroke care drawn from a survey of carers[5], shows that carers may experience: anxiety (79%), frustration (84%), sleeping disturbances (60%), depression (56%), and stress (57%). Deterioration in the health and wellbeing of the carer has important implications on the outcomes of stroke survivors including: poorer rehabilitation outcomes; reduced quality of life; heightened levels of depression; greater risk of mortality; poorer treatment adherence; and increased likelihood of being placed into institutional care, which has important cost implications for the NHS[6 7].

The Care Act 2014 has placed a statutory responsibility on local authorities in England to consider the wellbeing of carers as being of equal importance to the wellbeing of the people they care for[8]. The importance of providing support and intervention to carers has also been emphasised in national stroke guidelines[9 10]. Consequently, it is becoming more urgent to develop appropriate and effective interventions to meet the specific needs of carers of stroke survivors. However, little evidence exists with regard to which might be the most useful interventions for carers. This lack of evidence is not due to the lack of research in this field[11]; interventions directed at both stroke survivors and carers forms the largest body of research and has predominantly focused upon examining new models of service delivery[12], such as care-giver training, the stroke family support worker[13 14], and multidisciplinary hospital and community stroke teams. These interventions however have predominantly focused upon the

For peer review only - http://bmjopen.bmj.com/site/about/guidelines.xhtml

stroke survivors needs rather than the carers needs[15], and thus the needs of carers have largely been neglected. The few interventions to date that have been developed specifically for carers include education and information[16], skills training[17] and social support[18]. Such interventions however have produced inconclusive findings, arguably because such interventions are failing to address and meet the specific needs of carers. Forster et al[19] evaluated a structured caregiver training programme delivered in hospital by multidisciplinary teams from stroke units. There was no difference between the intervention and usual care and they concluded that the immediate period after stroke might not be the best time to deliver such a programme.

Given the high prevalence of psychological morbidity within the stroke carer population, there is likely to be a high demand for psychologically informed interventions targeted at informal carers of stroke survivors beyond the initial period of hospitalisation. Although evidence-based treatments for psychological difficulties exists, the associated costs and expenses of service delivery are high, with demand for treatment exceeding service capacity, resulting in long waiting lists[20] and limited access[21]. There are other barriers experienced by carers wishing to pursue and access mental healthservices[22]. These barriers include a lack of attention by health professionals of the difficulties associated with the caregiving role, and that general practitioners are often more likely to offer practical rather than psychological support. Together these reasons make it increasingly difficult for informal carers to access evidence-based psychologically informed interventions.

The biopsychosocial model of health and illness, as proposed by Engel[23], suggests that psychobiological vulnerability is influenced by an interaction of biological (physical health), psychological (thoughts, emotions and behaviours) and social (relationships and roles, activities) factors. The model emphasises the need for interventions to focus on both symptom reduction and on relapse prevention[24]. Psychological models such as cognitive-behavioural and interpersonal therapy have been deemed too fragmented and reductionist[25], given that they do not integrate the biological and psychological factors, as well as social, environmental and stress factors that are known to interfere with psychological functioning.

There have been movements towards the use of biopsychosocial interventions for the treatment of psychological difficulties amongst the general population. However, evidence suggests that significant adaptations to such interventions are required prior to application to different clinical populations. Indeed, mental health services for

For peer review only - http://bmjopen.bmj.com/site/about/guidelines.xhtml

carers have been criticised for not being tailored to address the unique and specific difficulties experienced by stroke carers[26]. Such difficulties can include having to manage the physical and cognitive impairment and behavioural difficulties the stroke survivor maybe presenting with[27]. There is growing recognition of the importance of understanding carer's experiences when dealing with health resources and healthcare policy[28].

Reflecting this recommendation, and as part of this research study, we developed a new biopsychosocial intervention, specifically targeted at informal carers of stroke survivors. The intervention was developed collaboratively with stroke carers and designed to be delivered in a group format to offer participants the opportunity to meet and interact with people and listen to how others have coped. Delivering the intervention in a group format is also likely to be more time and cost efficient, which would be important given the current demand for psychological therapies.

This study is examining the feasibility of conducting a randomised controlled trial (RCT) to examine the effectiveness and acceptability of this biopsychosocial intervention for stroke carers in the first year post-stroke.

Research Aim and Objectives

The ultimate aim of this study is to evaluate whether a biopsychosocial intervention can improve psychological outcomes in carers of stroke survivors (in the one year post-stroke period). However, we are not able to complete a definitive, powered trial until we have collected further information to inform the design of such a study. The purpose of this feasibility trial is to explore whether the biopsychosocial intervention for carers of stroke survivors is feasible, acceptable and to estimate the parameters for conducting a fully powered trial.

Primary Objective

The primary objective of this feasibility trial is to evaluate whether it is feasible to deliver a biopsychosocial intervention to carers of stroke survivors as part of a randomised controlled trial (RCT).

Secondary Objectives

This feasibility RCT will test the integrity of the study protocol, such as the methods of data collection, randomisation procedures and the masking of independent assessors.

For peer review only - http://bmjopen.bmj.com/site/about/guidelines.xhtml

This feasibility study will answer the necessary questions to inform a definitive multi-centre trial which include:

- Can we identify participants willing to be randomised?
- Can we deliver the intervention as planned?
- Is the intervention acceptable to participants?
- Can we retain participants in the study?
- What are the most relevant outcome measures?
- What is the consent rate?

METHODS AND ANALYSIS

Study Design and Setting

This is a single centre feasibility RCT with nested qualitative interview study. The RCT is a parallel group, two arm trial with a 1:1 allocation ratio biopsychosocial intervention: usual care control.

Participants

Participants are dyads of people who have had a stroke (stroke survivors) and their carers. Our definition of a carer is a family member or friend who is/will be providing support for a stroke survivor who would not be able to manage without their help due to their condition. Dyads will be recruited from stroke units at a University Hospital, community stroke services, and third sector stroke clubs and support groups.

The inclusion criteria are as follows:

Stroke survivors:

- Aged 18 or over
- Confirmed diagnosis of stroke
- Within one year of stroke onset
- Capacity to provide informed consent or consultee opinion that the person would wish to participate

Stroke carers:

- Aged 18 or over
- Carer of a person with a confirmed diagnosis of stroke within one year of stroke onset
- Capacity to provide informed consent
- Willing to attend a 6 week group intervention programme

For peer review only

The exclusion criteria are as follows:

Stroke survivors:

- Unable to speak English
- People engaged in other research involving biopsychosocial/psychological interventions

Stroke carers:

- Unable to speak English
- Engaged in other research involving biopsychosocial/psychological interventions
- People with visual (blindness) or auditory (deafness) impairments that would preclude them from participating in the therapy sessions.

Intervention Development

The intervention was developed based on the biopsychosocial model of health and illness[23] with the aim to address biological, psychological and social factors and symptom reduction and relapse prevention. The content was informed through a series of focus groups conducted with carers. Thematic analysis of focus group data revealed specific difficulties and challenges experienced by carers in the early post-stroke aftermath, and helpful coping strategies commonly used. We also conducted a nominal group approach with stroke rehabilitation experts to further refine the intervention. The biopsychosocial intervention was designed to recognise and target the difficulties commonly experienced by informal carers (identified through the focus groups and from the stroke literature). Additionally, helpful coping strategies used by the informal carers were used to further inform and adapt the content of the intervention. More detailed information about the development of the intervention will be provided in a further publication.

Intervention and Comparator

Participants are randomised as dyads. Participants will be randomised to either:

- **Control Group: usual care.** Dyads randomised to the control group will receive the usual range of routine care and services available to them. They will not receive the biopsychosocial intervention.

- **Intervention Group: Biopsychosocial Intervention, plus usual care**

For peer review only - http://bmjopen.bmj.com/site/about/guidelines.xhtml

For dyads randomised to the intervention group, the carer will receive the biopsychosocial intervention, in addition to usual care. The stroke carers randomised to receive the intervention will receive a two-hour session once a week for six weeks. The time-point at which the intervention will start will be agreed with the carer, in conjunction with other carers likely to be part of that group. This will occur when the stroke survivor they care for has been discharged from hospital, up to one year post-stroke. We will aim to deliver the intervention to groups of approximately 5 people. However in the event that it is not possible to coordinate sufficient people, or where carers are unable to attend the group sessions, we will deliver the intervention on a one to one basis or in smaller groups. We will record this as part of our feasibility. The intervention will be delivered at a suitable venue, with sufficient space and access for carer group members. The intervention sessions will be facilitated by a research psychologist who has received accredited training in the principles of biopsychosocial theory as well as specific training from members of the research team responsible for the development of the intervention. Clinical supervision and debriefing sessions will be provided by an experienced community mental health nurse with significant Community Stroke Team experience, and/or a clinical psychologist. Each session will last approximately 2 hours and will include a 15-minute tea/coffee break which will allow participants to interact more informally with one another, and the session will conclude with a 15-minute relaxation exercise. We anticipate that in the definitive trial, the intervention could be delivered by assistant psychologists with supervision from clinical psychologists.

The intervention programme is focused on adjustment to stroke, provision of psychoeducation and psychological support. The group programme is based on the principles of biopsychosocial model. The sessions are designed to teach individuals to identify and use skills to reduce current and future distress, thus aiding coping and adjustment to the impact of stroke and their role as a carer. The sessions are also intended to increase awareness of the role of thoughts, emotions and behaviours and their influence on each other. By practising problem solving and stress-management strategies, it is hoped that carers will experience fewer difficulties with their mood in the future.

For each session there will be a presentation containing information about a topic and exercises to aid discussion. Sessions will be presented on Microsoft

For peer review only - http://bmjopen.bmj.com/site/about/guidelines.xhtml

PowerPoint and all participants will receive either electronic or paper copies of the slides as appropriate, accompanied by the exercise and an in between session task. The topics will cover, for example, an introduction to stroke and caring, adjustment and mood, how to handle negative emotions and thoughts, dealing with problems, and a well-being relapse prevention plan. The content of these sessions was informed by the findings of earlier work described above. Relaxation exercises at the end of each session will allow participants to feel calm and relaxed before finishing their session, and also an effective tool for them to use outside of the session when experiencing high levels of anxiety or distress. Between session tasks will be set to encourage participants to practice exercises from the sessions in their own time. Participants who are identified to be experiencing significant issues that out with the scope of the intervention will be referred onto the appropriate specialist service, subject to their consent.

Outcomes

The main outcome for the study is to determine the feasibility of conducting a larger, powered study. This will be a composite of: whether the eligibility criteria are realistic; whether stroke survivors and carers are willing to be randomised; the study attrition rate; the feasibility and acceptability of delivering the intervention; the suitability and sensitivity of outcome measures; the most suitable outcome measure for use in the main study.

The stroke carer outcomes to be assessed at six months post-randomisation, will be: anxiety and depression; health related quality of life; and carer strain. The outcome measures which will be used are: Hospital Anxiety and Depression Scale[29]; EuroQol EQ5D-5L[30], and the caregiver burden scale[31].

The stroke survivor outcomes to be assessed six months post-randomisation, will be: level of disability; ability to perform personal activities of daily living; level of anxiety and depression; health related quality of life. The outcomes measures which will be used are: Modified Rankin Scale[32]; Barthel Index[33]; Hospital Anxiety and Depression Scale[29]; EuroQol EQ5D-5L[30]. The timeline and proposed flow of participants through the study is shown in Figure 1.

Feedback interviews

Qualitative semi-structured interviews will be conducted with carers in both trial arms of the study, within two weeks of their final outcome assessment. Up to 10 interviews will

For peer review only - http://bmjopen.bmj.com/site/about/guidelines.xhtml

For peer review only

be completed with stroke carers in each arm of the study. Our aim is to obtain feedback on all aspects of the study in addition to the intervention procedures, assessments, intervention (if received) and perceived outcomes. For those in the control group the interviews will provide confirmation of the nature of usual care received. Participants will be purposefully selected to include carers of stroke survivors with varying severity of stroke, age and gender. The interviews will be conducted by a researcher who had no involvement in the intervention, thereby reducing social desirability response bias. The researcher conducting the interviews will become aware of the group allocation during the interview and so will not be masked to the intervention. These interviews will be audio recorded using a digital recorder, transcribed and analysed using a thematic analysis (following the procedure described by Braun and Clarke[34]). The interviews with participants will provide information feedback on their perception of progress over time and for those in the intervention group, the quality of the intervention provided, and as such will serve as a process measure. Insights from the qualitative data and analysis will serve to inform developments of the intervention programme in the future and to generate user-orientated proposals about areas for further investigations. This information will also inform us of any refinements to be made to the study procedures. An interview will also be conducted with the group facilitator after they have completed all therapy. This interview will ask about the ease of delivery of the intervention according to the manual and any challenges.

Sample Size, Recruitment Strategy, Randomisation and Blinding

For a feasibility study, no formal sample size calculation is required. The aim is to recruit up to 40 dyads (20 in each arm of the trial) to test the randomisation process and the feasibility of the study processes of delivering the intervention. This target should allow us to collect sufficient information on the suitability and sensitivity of the outcome measures for use with this population and the standard deviations of the measures to inform a sample size calculation for a definitive trial. The median sample size for UK feasibility trials has been reported at 36[35] which is broadly consistent with the planned target.

The trial opened for recruitment on 1st November 2015 and will close on 31st July 2017 or when 40 dyads have been recruited (whichever is soonest). Participants will be enrolled into the study by a member of staff from the Clinical Research Network (CRN) or a member of the research team. The process for obtaining participant informed consent will be in accordance with the Research Ethics Committee (REC) guidance, and Good Clinical Practice (GCP) and any other regulatory requirements that might be introduced.

For peer review only - http://bmjopen.bmj.com/site/about/guidelines.xhtml

Following a full explanation of the study, the participant will be required to provide informed written consent before they can participate. Where a consultee is required for a stroke survivor, the consultee shall provide a recommendation as to whether they consider the person would have agreed to take part in the study, had they still had capacity to state their own preference. They will sign the consultee declaration, should they believe that person would have wished to take part in the study.

Randomisation to each group will be on a 1:1 basis, intervention:control. A simple randomisation procedure will be provided and overseen by the East Midlands Research Design Service (RDS). A researcher from the RDS, who is not otherwise involved in the study, will generate the randomisation sequence using computer generated random numbers and place allocations into sequentially numbered opaque, sealed envelopes. These will be accessed by the research team following consent and completion of the baseline assessment. The group facilitator will be informed of group allocation as they will be providing the treatment. We will take every step to minimise allocation and outcome bias.

Trial participants will not be masked to group allocation because they will need to be informed as to whether they have been allocated to the intervention group receiving the biopsychosocial intervention, or the control group. The participants' names, trial identifier numbers and treatment allocation will be stored on a password protected database held by the group facilitator. This database will be used to allow treatment allocations to be identified at the end of the study.

Baseline data will be collected and baseline assessments will be completed prior to randomisation. Baseline information will include:

1. Demographic details including age, gender, ethnicity and employment
2. Number and percentage of participants who meet eligibility criteria
3. Number of eligible participants who give consent
4. Levels of anxiety and depression (Hospital Anxiety and Depression Scale)
5. Quality of Life (EuroQol)
6. Carer strain (Carer Burden Scale).

In addition, we will collect the following information from the stroke survivor and/or their medical notes (with consent):

1. Stroke characteristics
2. Language and Cognitive Abilities (Montreal Cognitive Assessment)

For peer review only

For peer review only - http://bmjopen.bmj.com/site/about/guidelines.xhtml

3. Personal Activities of Daily Living (Barthel Index)
4. Stroke severity (National Institute of Health Stroke Scale)
5. Quality of Life (EuroQol)
6. Which service (if any) the stroke survivor is discharged to (e.g. ESD, intermediate care).

Follow-up assessment visits will be completed at six-months post-randomisation by a research assistant who is masked to allocation. To minimize the risk of unmasking, prior to each contact, the participant will be reminded that the researcher who is to conduct their follow-up assessment is masked. It is possible that participants may reveal their group allocation to the outcome assessors and any instances of this will be recorded by researchers as part of the assessment of feasibility; researchers will also be asked to make their 'best guess' as to the group allocation of the participants to determine whether masking was successful. Other members of the research team and investigators will not be masked to group allocation for the purpose of managing the trial and delivering the interventions. It will not be possible to mask participants.

Data Collection, Management and Analysis

Data will be collected on a paper case report form (CRF) and will subsequently be entered onto a secure password protected, purposely designed electronic database. Each participant will be assigned a trial identity code number, allocated at randomisation, for use on CRFs other trial documents and the electronic database to ensure confidentiality. The documents and database will also use their initials and date of birth. CRFs will be treated as confidential documents and held securely in accordance with regulations. Only the research team and sponsor will have access to the data. Data will be analysed by the research team.

When data collection is complete, a data quality check will be conducted in duplicate by two researchers and a 10% sample of the database will be checked against the original paper CRF. Steps will be taken to minimise missing data by personal contact throughout the study period from the investigator and every attempt will be made to locate participants for follow-up. Outcome data will be collected in person, in the participant's home, by a research assistant to minimise the amount of missing data. For each outcome measure used where data is missing, an imputed average will be used for items where less than 10% of the overall measure is missing. Where more than 10% of a measure is missing, the entire measure will be coded as missing, unless the scoring criterion for that measure stipulates an alternative approach. We will not collect any

For peer review only - http://bmjopen.bmj.com/site/about/guidelines.xhtml

further data for participants who withdraw from the study, but we will retain all data collected up until the point of withdrawal.

The following procedures will apply to data analysis:

Acceptability of the study design

Recruitment rates, proportion of carers screened who are eligible for enrolment and who provided consent, how easily carers can be identified, who met the criteria for the study, number of people who accepted intervention to take part in the RCT, number of individuals who attended the intervention/number of sessions they attended. The feedback interviews will provide further information regarding the acceptability of the intervention. Qualitative thematic analysis will provide an insight into carer perspectives of their experience of caring and what effect they think the intervention itself may have had (for the treatment group).

Feasibility of completing the intervention

Proportion of carers completing the assessment and interventions. Feedback interviews will also provide information about delivery of the intervention both from the perspective of the group facilitator and the experiences of the carers themselves.

Tolerability

Proportion of carers who withdraw or decline intervention. Record of interventions declined and why.

Integrity of the study protocol

By examining how many participants are able to complete the study, % of missing data, percentage of people who completed questionnaires, percentage of people who completed each outcome measures at 6 month follow up, calculation of the cost of running the study.

Outcome measures

Outcome measure data will be stored in a database and data will be analysed using the statistical package STATA. The proportion of missing items will be examined. The questionnaire data will be analysed to determine the distributions of scores. The analysis will use descriptive statistics and confidence intervals for the parameters we are estimating. The characteristics of stroke survivors and their carers will also be described using means, standard deviations and ranges for quantitative variables and counts and

For peer review only - http://bmjopen.bmj.com/site/about/guidelines.xhtml

proportions for categorical variables. Data will be analysed on an 'intent to treat' basis. Any changes in the planned statistical methods will be documented in the report.

Ethics

Ethical approval for this study was provided by East Midlands – Nottingham 2 Research Ethics Committee (14/EMI/1264). Health Research Authority (HRA) and research and development approvals have been obtained as necessary.

DISSEMINATION

This study will provide the foundations and information needed to inform a further, appropriately powered study to investigate the effectiveness of the biopsychosocial intervention for stroke survivors and their carers. There are few interventional studies for stroke carers and this study is addressing a key area of concern for the stroke community. The findings will therefore be relevant to researchers, clinicians, commissioners, stroke survivors and carers. Additionally, there is an increasing focus on interventions which prevent or delay the need for other health and social care services; the findings will also be relevant to policymakers in this area.

We plan to disseminate our findings through presentations at national and international stroke and rehabilitation conferences, and we will submit findings for publication in a peer reviewed academic journal.

Trial Status

The trial is in recruitment phase. Recruitment commenced in November 2015; recruitment is due to close in July 2017. The trial is registered ISRCTN 15643456.

List of Abbreviations

BISC – Biopsychosocial Intervention for Stroke Carers

CRF – Case report form

CRN – Clinical Research Network

GCP – Good Clinical Practice

HRA – Health Research Authority

RCT – Randomised Controlled Trial

RDS – Research Design Service

REC – Research Ethics Committee

Competing Interests

For peer review only

For peer review only - http://bmjopen.bmj.com/site/about/guidelines.xhtml

None declared.

Authors' Contributions

MW, ST, PW, RF and CC drafted the manuscript. MW, RF and CC conceived the study, MW is the principal investigator. LC contributed to the design of the study and delivers the intervention. All authors are members of the research team involved in the running of the study. All authors commented critically on the manuscript and read and approved the final manuscript.

Acknowledgements

We would like to thank the project steering group: Sheila Birchall, Charlotte Davies, Christopher Greensmith, Kate Hooban, Ben Jackson, Raj Mehta and Nikola Sprigg. We would also like to thank Joanna Fletcher-Smith, Miriam Golding-Day, Eirini Kontou, Oliver Matias and Helen Taylor for their assistance with conducing the study.

Study Sponsor and Funder

The sponsor for this study is the University of Nottingham, Research and Graduate Services, King's Meadow Campus, Lenton Lane, Nottingham, NG7 2NR.

This protocol is independent research funded by the National Institute for Health Research (Research for Patient Benefit Programme, Biopsychosocial Intervention for Stroke Carers (BISC), PB-PG-0613-31064). The views expressed in this publication are those of the authors and not necessarily those of the NHS, the National Institute for Health Research or the Department of Health.

For peer review only

References

1. Scottish Government Health Directorate. National Framework for Service Change in the NHS in Scotland. Self care, carers, volunteering and the voluntary sector: towards a more collaborative approach. Secondary National Framework for Service Change in the NHS in Scotland. Self care, carers, volunteering and the voluntary sector: towards a more collaborative approach 2005. http://www.sehd.scot.nhs.uk/NationalFramework/Documents/electivecare/Selfcare230505.pdf.

2. McCullagh E, Brigstocke G, Donaldson N, et al. Determinants of caregiving burden and quality of life in caregivers of stroke patients. Stroke (00392499) 2005;**36**(10):2181-86.

3. Han B, Haley WE. Family caregiving for patients with stroke. Review and analysis. Stroke 1999;**30**(7):1478-85.

4. Brereton L. Preparation for family care-giving: stroke as a paradigm case. Journal of clinical nursing 1997;**6**(6):425-34.

5. The Stroke Association. Feeling Overwhelmed: The emotional impact of stroke. Life After Stroke Compaign Report. London: The Stroke Association, 2013.

6. Carnwath T, Johnson D. Psychiatric morbidity among spouses of patients with stroke. Br Med J (Clin Res Ed) 1987;**294**(6569):409-11.

7. Evans RL, Matlock AL, Bishop DS, et al. Family intervention after stroke: Does counseling or education help? Stroke 1988;**19**(10):1243-49.

8. Department of Health. Care and Support statutory guidance. Secondary Care and Support statutory guidance 2017. https://www.gov.uk/government/publications/care-act-statutory-guidance/care-and-support-statutory-guidance#fn:1.

9. Intercollegiate Stroke Working Party. National clinical guideline for stroke. Fifth ed. London: Royal College of Physicians, 2016.

10. National Institute for Health and Care Excellence. Stroke Rehabilitation in Adults. London: National Institute for Health and Care Excellence, 2013.

For peer review only

For peer review only - http://bmjopen.bmj.com/site/about/guidelines.xhtml

11. Legg LA, Quinn TJ, Mahmood F, et al. Non-pharmacological interventions for caregivers of stroke survivors. Cochrane Database of Systematic Reviews 2011(10):CD008179.

12. Anderson C, Mhurchu C, Rubenach S, et al. Hospital or home for stroke rehabilitation? Results of a randomised controlled trial. II: cost minimization analysis at 6 months. Stroke 2000;**31**:1032-37.

13. Lincoln NB, Francis VM, Lilley SA, et al. Evaluation of a stroke family support organiser: a randomized controlled trial. Stroke 2003;**34**(1):116-21.

14. Ellis G, Mant J, Langhorne P, et al. Stroke liaison workers for stroke patients and carers: an individual patient data meta-analysis. Cochrane Database of Systematic Reviews 2010(5):CD005066.

15. Greenwood N, Mackenzie A, Harris R, et al. Perceptions of the role of general practice and practical support measures for carers of stroke survivors: a qualitative study. BMC family practice 2011;**12**(1):57.

16. Smith LN, Lawrence M, Kerr SM, et al. Informal carers' experience of caring for stroke survivors. Journal of Advanced Nursing 2004;**46**(3):235-44.

17. Patel A, Knapp M, Evans A, et al. Training care givers of stroke patients: Economic evaluation. BMJ: British Medical Journal 2004;**328**(7448):1102.

18. Printz-Feddersen V. Group process effect on caregiver burden. Journal of Neuroscience Nursing 1990;**22**(3):164-68.

19. Forster A, Dickerson J, Young J, et al. A structured training programme for caregivers of inpatients after stroke (TRACS): a cluster randomised controlled trial and cost-effectiveness analysis. Lancet 2013;**382**(9910):2069-76.

20. Lovell K, Richards DA, Bower P. Improving access to primary mental health care: uncontrolled evaluation of a pilot self-help clinic. Br J Gen Pract 2003;**53**(487):133-35.

21. Kazdin AE, Blase SL. Rebooting psychotherapy research and practice to reduce the burden of mental illness. Perspectives on psychological science 2011;**6**(1):21-37.

For peer review only

For peer review only - http://bmjopen.bmj.com/site/about/guidelines.xhtml

22. Arksey H, Hirst M. Unpaid carers' access to and use of primary care services. Primary Health Care Research and Development 2005;**6**(02):101-16.

23. Engel GL. The need for a new medical model: a challenge for biomedicine. Science 1977;**196**(4286):129-36.

24. Engel GL. The clinical application of the biopsychosocial model. Am J Psychiatry 1980;**137**(5):535-44.

25. Schotte CK, Van Den Bossche B, De Doncker D, et al. A biopsychosocial model as a guide for psychoeducation and treatment of depression. Depression and anxiety 2006;**23**(5):312-24.

26. Lundh U. Family carers 4: designing services to support family carers in Sweden. British Journal of Nursing 1999;**8**(12):787-90.

27. van den Heuvel ET, de Witte LP, Schure LM, et al. Risk factors for burn-out in caregivers of stroke patients, and possibilities for intervention. Clinical Rehabilitation 2001;**15**(6):669-77.

28. Nilsen ES, Myrhaug HT, Johansen M, et al. Methods of consumer involvement in developing healthcare policy and research, clinical practice guidelines and patient information material. Cochrane Database Syst Rev 2006;**3**(3).

29. Zigmond AS, Snaith RP. The hospital anxiety and depression scale. Acta psychiatrica scandinavica 1983;**67**(6):361-70.

30. EuroQol Group. EuroQol – a new facility for the measurement of health-related quality of life. Health Policy 1990;**16**:199-208.

31. Zarit SH, Reever KE, Bach-Peterson J. Relatives of the Impaired Elderly: Correlates of Feelings of Burden. The Gerontologist 1980;**20**(6):649-55.

32. Bonita R, Beaglehole R. Modification of Rankin Scale: Recovery of motor function after stroke. Stroke 1988;**19**(12):1497-500.

33. Collin C, Wade D, Davies S, et al. The Barthel ADL Index: a reliability study. Disability & Rehabilitation 1988;**10**(2):61-63.

For peer review only

For peer review only - http://bmjopen.bmj.com/site/about/guidelines.xhtml

34. Braun V, Clarke V. Using thematic analysis in psychology. Qualitative Research in Psychology 2006;**3**(2):77-101.

35. Billingham SA, Whitehead AL, Julious SA. An audit of sample sizes for pilot and feasibility trials being undertaken in the United Kingdom registered in the United Kingdom Clinical Research Network database. BMC medical research methodology 2013;**13**(1):1.

For peer review only

For peer review only - http://bmjopen.bmj.com/site/about/guidelines.xhtml

Figure 1: FLOW of Participants through the study

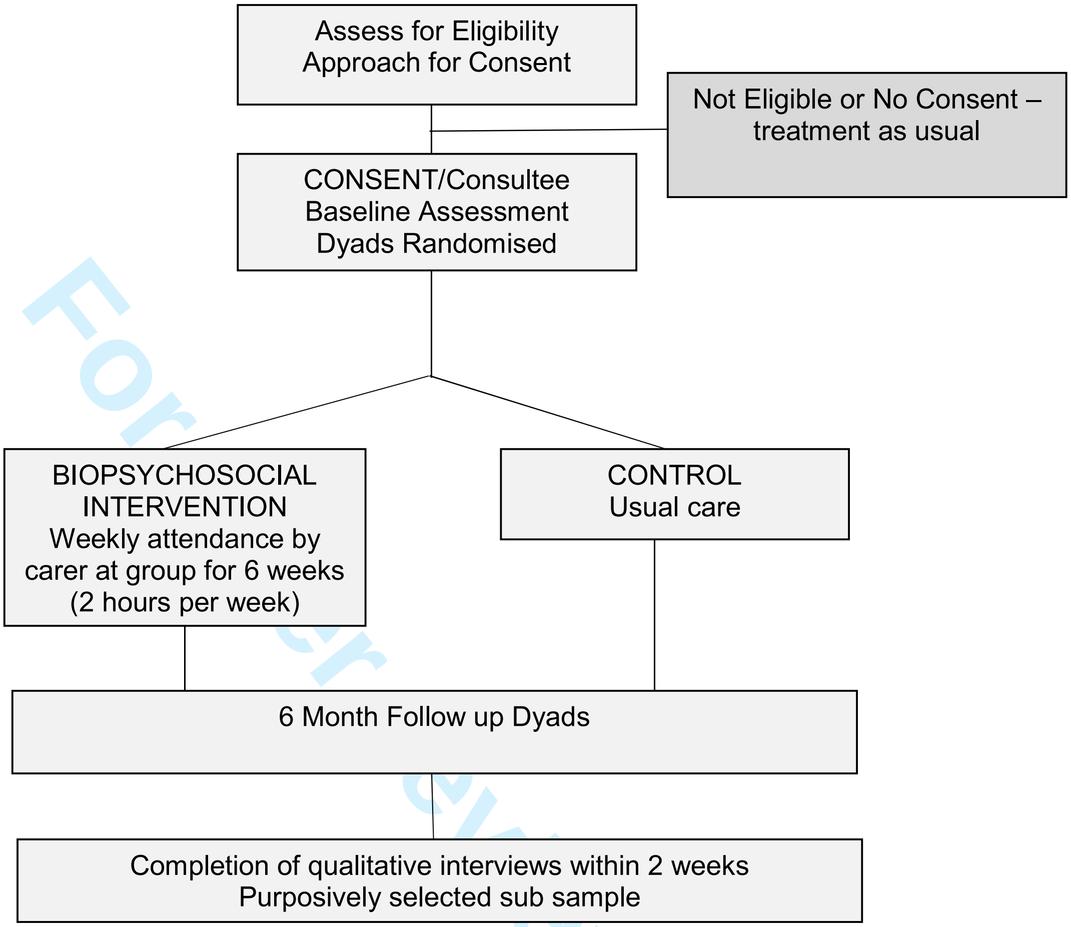

For peer review only - http://bmjopen.bmj.com/site/about/guidelines.xhtml

12

STANDARD PROTOCOL ITEMS: RECOMMENDATIONS FOR INTERVENTIONAL TRIALS

For peer review only

SPIRIT 2013 Checklist: Recommended items to address in a clinical trial protocol and related documents*

Section/item	Item No	Description	Addressed on page number
**Administrative information**			
Title	1	Descriptive title identifying the study design, population, interventions, and, if applicable, trial acronym	___ 1___
Trial registration	2a	Trial identifier and registry name. If not yet registered, name of intended registry	_________1_____
	2b	All items from the World Health Organization Trial Registration Data Set	___Throughout__
Protocol version	3	Date and version identifier	_________1_____
Funding	4	Sources and types of financial, material, and other support	________16______
Roles and responsibilities	5a	Names, affiliations, and roles of protocol contributors	_____1 & 15____
	5b	Name and contact information for the trial sponsor	________16______
	5c	Role of study sponsor and funders, if any, in study design; collection, management, analysis, and interpretation of data; writing of the report; and the decision to submit the report for publication, including whether they will have ultimate authority over an4 - 6y of these activities	________16______
	5d	Composition, roles, and responsibilities of the coordinating centre, steering committee, endpoint adjudication committee, data management team, and other individuals or groups overseeing the trial, if applicable (see Item 21a for data monitoring committee)	_______N/A______

For peer review only - http://bmjopen.bmj.com/site/about/guidelines.xhtml

Introduction

Background and rationale	6a	Description of research question and justification for undertaking the trial, including summary of relevant studies (published and unpublished) examining benefits and harms for each intervention	________4-6 ______
	6b	Explanation for choice of comparators	______8________
Objectives	7	Specific objectives or hypotheses	______6-7_______
Trial design	8	Description of trial design including type of trial (eg, parallel group, crossover, factorial, single group), allocation ratio, and framework (eg, superiority, equivalence, noninferiority, exploratory)	________7_______

Methods: Participants, interventions, and outcomes

Study setting	9	Description of study settings (eg, community clinic, academic hospital) and list of countries where data will be collected. Reference to where list of study sites can be obtained	_________7______
Eligibility criteria	10	Inclusion and exclusion criteria for participants. If applicable, eligibility criteria for study centres and individuals who will perform the interventions (eg, surgeons, psychotherapists)	________7-8______
Interventions	11a	Interventions for each group with sufficient detail to allow replication, including how and when they will be administered	________8-10_____
	11b	Criteria for discontinuing or modifying allocated interventions for a given trial participant (eg, drug dose change in response to harms, participant request, or improving/worsening disease)	________9_______
	11c	Strategies to improve adherence to intervention protocols, and any procedures for monitoring adherence (eg, drug tablet return, laboratory tests)	_______10 &14____
	11d	Relevant concomitant care and interventions that are permitted or prohibited during the trial	_______8________
Outcomes	12	Primary, secondary, and other outcomes, including the specific measurement variable (eg, systolic blood pressure), analysis metric (eg, change from baseline, final value, time to event), method of aggregation (eg, median, proportion), and time point for each outcome. Explanation of the clinical relevance of chosen efficacy and harm outcomes is strongly recommended	________10_______
Participant timeline	13	Time schedule of enrolment, interventions (including any run-ins and washouts), assessments, and visits for participants. A schematic diagram is highly recommended (see Figure)	Figure 1 & 8-9

For peer review only - http://bmjopen.bmj.com/site/about/guidelines.xhtml

Sample size	14	Estimated number of participants needed to achieve study objectives and how it was determined, including clinical and statistical assumptions supporting any sample size calculations	______11_______
Recruitment	15	Strategies for achieving adequate participant enrolment to reach target sample size	_______11 -12___
**Methods: Assignment of interventions (for controlled trials)**			
Allocation:			
Sequence generation	16a	Method of generating the allocation sequence (eg, computer-generated random numbers), and list of any factors for stratification. To reduce predictability of a random sequence, details of any planned restriction (eg, blocking) should be provided in a separate document that is unavailable to those who enrol participants or assign interventions	______12_______
Allocation concealment mechanism	16b	Mechanism of implementing the allocation sequence (eg, central telephone; sequentially numbered, opaque, sealed envelopes), describing any steps to conceal the sequence until interventions are assigned	_______12______
Implementation	16c	Who will generate the allocation sequence, who will enrol participants, and who will assign participants to interventions	________12_____
Blinding (masking)	17a	Who will be blinded after assignment to interventions (eg, trial participants, care providers, outcome assessors, data analysts), and how	_______12-13 ___
	17b	If blinded, circumstances under which unblinding is permissible, and procedure for revealing a participant's allocated intervention during the trial	________N/A____
**Methods: Data collection, management, and analysis**			
Data collection methods	18a	Plans for assessment and collection of outcome, baseline, and other trial data, including any related processes to promote data quality (eg, duplicate measurements, training of assessors) and a description of study instruments (eg, questionnaires, laboratory tests) along with their reliability and validity, if known. Reference to where data collection forms can be found, if not in the protocol	________13_____
	18b	Plans to promote participant retention and complete follow-up, including list of any outcome data to be collected for participants who discontinue or deviate from intervention protocols	_______13______

Data management	19	Plans for data entry, coding, security, and storage, including any related processes to promote data quality (eg, double data entry; range checks for data values). Reference to where details of data management procedures can be found, if not in the protocol	_______13_______
Statistical methods	20a	Statistical methods for analysing primary and secondary outcomes. Reference to where other details of the statistical analysis plan can be found, if not in the protocol	________13-14 ___
	20b	Methods for any additional analyses (eg, subgroup and adjusted analyses)	__________N/A___
	20c	Definition of analysis population relating to protocol non-adherence (eg, as randomised analysis), and any statistical methods to handle missing data (eg, multiple imputation)	________13______
**Methods: Monitoring**			
Data monitoring	21a	Composition of data monitoring committee (DMC); summary of its role and reporting structure; statement of whether it is independent from the sponsor and competing interests; and reference to where further details about its charter can be found, if not in the protocol. Alternatively, an explanation of why a DMC is not needed	__________N/A___
	21b	Description of any interim analyses and stopping guidelines, including who will have access to these interim results and make the final decision to terminate the trial	_______N/A______
Harms	22	Plans for collecting, assessing, reporting, and managing solicited and spontaneously reported adverse events and other unintended effects of trial interventions or trial conduct	_______N/A______
Auditing	23	Frequency and procedures for auditing trial conduct, if any, and whether the process will be independent from investigators and the sponsor	________N/A_____
**Ethics and dissemination**			
Research ethics approval	24	Plans for seeking research ethics committee/institutional review board (REC/IRB) approval	_________14____
Protocol amendments	25	Plans for communicating important protocol modifications (eg, changes to eligibility criteria, outcomes, analyses) to relevant parties (eg, investigators, REC/IRBs, trial participants, trial registries, journals, regulators)	_________N/A____

For peer review only - http://bmjopen.bmj.com/site/about/guidelines.xhtml

Consent or assent	26a	Who will obtain informed consent or assent from potential trial participants or authorised surrogates, and how (see Item 32)	________11______
	26b	Additional consent provisions for collection and use of participant data and biological specimens in ancillary studies, if applicable	_________N/A____
Confidentiality	27	How personal information about potential and enrolled participants will be collected, shared, and maintained in order to protect confidentiality before, during, and after the trial	________13______
Declaration of interests	28	Financial and other competing interests for principal investigators for the overall trial and each study site	_________15_____
Access to data	29	Statement of who will have access to the final trial dataset, and disclosure of contractual agreements that limit such access for investigators	________13______
Ancillary and post-trial care	30	Provisions, if any, for ancillary and post-trial care, and for compensation to those who suffer harm from trial participation	________N/A_____
Dissemination policy	31a	Plans for investigators and sponsor to communicate trial results to participants, healthcare professionals, the public, and other relevant groups (eg, via publication, reporting in results databases, or other data sharing arrangements), including any publication restrictions	________14-15___
	31b	Authorship eligibility guidelines and any intended use of professional writers	________15______
	31c	Plans, if any, for granting public access to the full protocol, participant-level dataset, and statistical code	________N/A_____
**Appendices**			
Informed consent materials	32	Model consent form and other related documentation given to participants and authorised surrogates	________N/A_____
Biological specimens	33	Plans for collection, laboratory evaluation, and storage of biological specimens for genetic or molecular analysis in the current trial and for future use in ancillary studies, if applicable	________N/A_____

*It is strongly recommended that this checklist be read in conjunction with the SPIRIT 2013 Explanation & Elaboration for important clarification on the items. Amendments to the protocol should be tracked and dated. The SPIRIT checklist is copyrighted by the SPIRIT Group under the Creative Commons "Attribution-NonCommercial-NoDerivs 3.0 Unported" license.

For peer review only - http://bmjopen.bmj.com/site/about/guidelines.xhtml

Biopsychosocial Intervention for Stroke Carers (BISC): Protocol for a Feasibility Randomised Controlled Trial (RCT)

Journal:	*BMJ Open*
Manuscript ID	bmjopen-2017-018309.R1
Article Type:	Protocol
Date Submitted by the Author:	21-Aug-2017
Complete List of Authors:	Walker, Marion; Faculty of Medicine & Health Sciences, University of Nottingham<br>Thomas, Shirley<br>Whitehead, Phillip; University of Nottingham, Division of Rehabilitation and Ageing<br>Condon, Laura<br>Fisher, Rebecca; Univ Nottingham<br>Kontou, Eirini; University of Nottingham, Division of Rehabilitation & Ageing<br>Benford, Penny; University of Nottingham, c/o Division of Rehabilitation & Ageing<br>Cobley, Christine; Derbyshire Healthcare NHS Foundation Trust
**Primary Subject Heading**:	Health services research
Secondary Subject Heading:	Rehabilitation medicine, Neurology
Keywords:	Carers, Stroke < NEUROLOGY, Biospsychosocial model

SCHOLARONE™ Manuscripts

Biopsychosocial Intervention for Stroke Carers (BISC): Protocol for a Feasibility Randomised Controlled Trial (RCT)

Marion F Walker*
Division of Rehabilitation and Ageing, University of Nottingham
B Floor, The Medical School, Queens Medical Centre, Nottingham, NG7 2UH
marion.walker@nottingham.ac.uk

Shirley A Thomas
Division of Rehabilitation and Ageing, University of Nottingham
B Floor, The Medical School, Queens Medical Centre, Nottingham, NG7 2UH
shirley.thomas@nottingham.ac.uk

Phillip J Whitehead
Division of Rehabilitation and Ageing, University of Nottingham
B Floor, The Medical School, Queens Medical Centre, Nottingham, NG7 2UH
phillip.j.whitehead@nottingham.ac.uk

Laura Condon
Division of Rehabilitation and Ageing, University of Nottingham
B Floor, The Medical School, Queens Medical Centre, Nottingham, NG7 2UH
laura.condon@nottingham.ac.uk

Rebecca Fisher
Division of Rehabilitation and Ageing, University of Nottingham
B Floor, The Medical School, Queens Medical Centre, Nottingham, NG7 2UH
rebecca.fisher@nottingham.ac.uk

Eirini Kontou
Division of Rehabilitation and Ageing, University of Nottingham
B Floor, The Medical School, Queens Medical Centre, Nottingham, NG7 2UH
eirini.kontou@nottingham.ac.uk

Penny Benford
Division of Rehabilitation and Ageing, University of Nottingham
B Floor, The Medical School, Queens Medical Centre, Nottingham, NG7 2UH
penny.benford@nottingham.ac.uk

Christine Cobley
Department of Clinical Psychology, Derbyshire Healthcare NHS Foundation Trust, St Andrews House, 201 London Road, Derby, DE1 2TZ.
Christine.cobley@derbyshcft.nhs.uk

*Corresponding Author

Word Count (main text): 4267

Protocol Version 5.0 17.10.16

For peer review only - http://bmjopen.bmj.com/site/about/guidelines.xhtml

Abstract

Introduction

Reducing length of hospital stay for stroke survivors often creates a shift in the responsibility of care towards informal carers. Adjustment to the caregiving process is experienced by many carers as overwhelming, complex and demanding, and can have a detrimental impact on mental and physical health and wellbeing. National policy guidelines recommend that carers' needs are considered and addressed; despite this, few interventions have been developed and empirically evaluated. We developed a biopsychosocial intervention in collaboration with carers of stroke survivors. Our aim is to determine whether the intervention can be delivered in a group setting and evaluated using a randomised controlled trial (RCT).

Methods and Analysis

Feasibility randomised controlled trial (RCT) and nested qualitative interview study. We aim to recruit up to 40 stroke carers within one year of the stroke onset. Carers are randomised to usual care or usual care plus biopsychosocial intervention. Each intervention group will consist of five stroke carers. The intervention will focus on: psychoeducation, psychological adjustment to stroke, strategies for reducing unwanted negative thoughts and emotions, and problem solving strategies. The main outcome is the feasibility of conducting an RCT. Carer outcomes at six months include: anxiety and depression, quality of life, and carer strain. Data is also collected from stroke survivors at baseline and six months including: level of disability, anxiety and depression, and quality of life.

Ethics and Dissemination

Favourable ethical opinion was provided by East Midlands – Nottingham2 Research Ethics Committee (14/EMI/1264). This study will determine whether delivery of the biopsychosocial intervention is feasible and acceptable to stroke carers within a group format. It will also determine whether it is feasible to evaluate the effects of the biopsychosocial intervention in an RCT. We will disseminate our findings through peer-reviewed publications and presentations at national and international conferences.

Trial Registration: ISRCTN15643456

For peer review only - http://bmjopen.bmj.com/site/about/guidelines.xhtml

Strengths and limitations of this study

- Enhancing the wellbeing of carers is a national priority. However, few interventions for carers have been developed or evaluated.
- This is a pragmatic trial conducted in a real world setting. The intervention content is based on the findings from the literature, developmental work with carers of stroke survivors and stroke rehabilitation experts.
- This feasibility study will be conducted in a single site only.
- The intervention focusses only on the initial stages of carer support (up to one year post-stroke onset). Significant problems may develop for carers at later stages which need to be identified and referred for more intensive/specialist support.

For peer review only

For peer review only - http://bmjopen.bmj.com/site/about/guidelines.xhtml

INTRODUCTION

Background and Rationale

A carer has been defined as "a person of any age who provides unpaid help and support to a relative, friend or neighbour who cannot manage to live independently without the carer's help due to frailty, illness, disability or addiction"[1]. Carers play a vital role in the early rehabilitation process and long-term management of the stroke survivor[2]. Carers deal with a range of care needs and demands including mobility, self-care, communication difficulties as well as cognitive impairment, mood and personality changes in the stroke survivor[3].

The latest figures from the Sentinel Stroke National Audit in England, Wales and Northern Ireland show that the median length of inpatient stay is between 7 and 8 days; however, just under one third of stroke survivors who were discharged requiring help with daily activities received assistance from informal carers[4]. This demonstrates that increasingly shorter hospital stays coincide with an earlier transfer of care to informal carers in the community. The Care Act 2014 has placed a statutory responsibility on local authorities in England to consider the wellbeing of carers as being of equal importance to the wellbeing of the people they care for[5]. The importance of providing support and intervention to carers has also been emphasised in national stroke guidelines[6 7]. Consequently, it is becoming more urgent to develop appropriate and effective interventions to meet the specific needs of carers of stroke survivors.

A growing number of people are unexpectedly finding themselves in the caregiving role. Although it can be a positive and rewarding role[8], the increased demands associated with informal caring can place carers at elevated risk of poorer mental and physical health, accompanied by reduced opportunity for paid employment and social interaction and activity[3]. An estimate of the psychosocial impact that might be associated with stroke care, drawn from a survey of carers[9], shows that carers may experience: anxiety (79%), frustration (84%), sleeping disturbances (60%), depression (56%), and stress (57%). Deterioration in the health and wellbeing of the carer has important implications on the outcomes of stroke survivors including: poorer rehabilitation outcomes; reduced quality of life; heightened levels of depression; greater risk of mortality; poorer treatment adherence; and increased likelihood of being placed into institutional care, which has important cost implications for the NHS, social care services, the stroke survivor and their family.

For peer review only

For peer review only - http://bmjopen.bmj.com/site/about/guidelines.xhtml

An increased focus on the needs of stroke carers has led to a spate of recent systematic reviews including quantitative and interventional studies[10-13], qualitative research[8 14] and economic evidence[15]. However, the results are equivocal and limited. Thus, there is no clear and robust evidence regarding the most effective and cost effective interventions for stroke carers. This lack of evidence is not due to the lack of research in this field[11]; interventions directed at both stroke survivors and carers forms the largest body of research and has predominantly focused upon examining new models of service delivery[16], such as care-giver training, the stroke family support worker[17 18], and multidisciplinary hospital and community stroke teams. These interventions however have predominantly focused upon the stroke survivors needs rather than the carers needs[19], and thus the needs of carers have largely been neglected. The few interventions to date that have been developed specifically for carers include education and information[20], skills training[21] and social support[22]. Such interventions however have produced inconclusive findings, arguably because such interventions are failing to address and meet the specific needs of carers. Forster et al[23] evaluated a structured caregiver training programme delivered in hospital by multidisciplinary teams from stroke units. There was no difference between the intervention and usual care and they concluded that the immediate period after stroke might not be the best time to deliver such a programme.

Given the high prevalence of psychological morbidity within the stroke carer population, there is likely to be a high demand for psychologically informed interventions targeted at informal carers of stroke survivors beyond the initial period of hospitalisation. Although evidence-based treatments for psychological difficulties exist, the associated costs and expenses of service delivery are high, with demand for treatment exceeding service capacity, resulting in long waiting lists[24] and limited access[25]. There are other barriers experienced by carers wishing to pursue and access mental health services[26]. These barriers include a lack of attention by health professionals of the difficulties associated with the caregiving role, and that general practitioners are often more likely to offer practical rather than psychological support. Together these reasons make it increasingly difficult for informal carers to access evidence-based psychologically informed interventions.

The biopsychosocial model of health and illness, as proposed by Engel[27], suggests that psychobiological vulnerability is influenced by an interaction of biological (physical health), psychological (thoughts, emotions and behaviours) and social (relationships

For peer review only - http://bmjopen.bmj.com/site/about/guidelines.xhtml

and roles) factors. The model emphasises the need for interventions to focus on both symptom reduction and on relapse prevention[28]. Psychological models such as cognitive-behavioural and interpersonal therapy have been deemed too fragmented and reductionist[29], given that they do not integrate the biological and psychological factors, as well as social, environmental and stress factors that are known to interfere with psychological functioning.

There have been movements towards the use of biopsychosocial interventions for the treatment of psychological difficulties amongst the general population. However, evidence suggests that significant adaptations to such interventions are required prior to application to different clinical populations. Indeed, mental health services for carers have been criticised for not being tailored to address the unique and specific difficulties experienced by stroke carers[30 31]. Such difficulties can include having to manage the physical and cognitive impairment and behavioural difficulties the stroke survivor may be presenting with[32 33]. There is growing recognition of the importance of understanding carer's experiences when dealing with health resources and healthcare policy[34]. A systematic review of psychosocial interventions for stroke carers concluded that more randomised controlled trials of psychoeducation programmes are needed[12].

Considering this recommendation in the context of the wider literature on stroke carers, we developed a new biopsychosocial intervention specifically targeted at informal carers of stroke survivors. The intervention was developed collaboratively with stroke carers and designed to be delivered in a group format to offer participants the opportunity to meet and interact with people and listen to how others have coped. Delivering the intervention in a group format is also likely to be more time and cost efficient, which would be important given the current demand for psychological therapies.

This study is examining the feasibility of conducting a randomised controlled trial (RCT) to examine the effectiveness and acceptability of this group biopsychosocial intervention for stroke carers in the first year post-stroke.

Research Aim and Objectives

The ultimate aim of this study is to evaluate whether a biopsychosocial intervention can improve psychological outcomes in carers of stroke survivors (in the one year post-stroke period). However, we are not able to complete a definitive, powered trial until we have collected further information to inform the design of such a study. The purpose of

For peer review only - http://bmjopen.bmj.com/site/about/guidelines.xhtml

this feasibility trial is to explore whether the biopsychosocial intervention for carers of stroke survivors is feasible, acceptable and to estimate the parameters for conducting a fully powered trial.

Primary Objective

The primary objective of this feasibility trial is to evaluate whether it is feasible to deliver a biopsychosocial intervention to carers of stroke survivors as part of a randomised controlled trial (RCT).

Secondary Objectives

This feasibility RCT will test the integrity of the study protocol, such as the methods of data collection, randomisation procedures and the masking of independent assessors. This feasibility study will answer the necessary questions to inform a definitive multi-centre trial which include:

- Can we identify participants willing to be randomised?
- Can we deliver the intervention as planned?
- Is the intervention acceptable to participants?
- Can we retain participants in the study?
- What are the most relevant outcome measures?
- What is the consent rate?

METHODS AND ANALYSIS

Study Design and Setting

This is a single centre feasibility RCT with nested qualitative interview study. The RCT is a parallel group, two arm trial with a 1:1 allocation ratio biopsychosocial intervention: usual care control.

Participants

Participants are carers of people who have had a stroke (stroke survivors). Our definition of a carer is a family member or friend who is/will be providing support for a stroke survivor who would not be able to manage without their help due to their condition. Carers will be recruited along with stroke survivors from stroke units at a University Hospital, community stroke services, and third sector stroke clubs and support groups. However, only the carer will receive the intervention; we will recruit the stroke survivor because we also aim to collect baseline and follow-up data from them.

The inclusion criteria are as follows:

For peer review only - http://bmjopen.bmj.com/site/about/guidelines.xhtml

Stroke carers:

- Aged 18 or over
- Carer of a person with a confirmed diagnosis of stroke within one year of stroke onset
- Capacity to provide informed consent
- Willing to attend a 6 week group intervention programme

Stroke survivors:

- Aged 18 or over
- Confirmed diagnosis of stroke
- Within one year of stroke onset
- Capacity to provide informed consent or consultee opinion that the person would wish to participate

The exclusion criteria are as follows:

Stroke carers:

- Unable to speak English
- Engaged in other research involving biopsychosocial/psychological interventions
- People with visual (blindness) or auditory (deafness) impairments that would preclude them from participating in the therapy sessions.

Stroke survivors:

- Unable to speak English
- People engaged in other research involving biopsychosocial/psychological interventions

Intervention Development

The intervention was developed based on the biopsychosocial model of health and illness[27] with the aim to address biological, psychological and social factors and symptom reduction and relapse prevention. The content was informed through a review of the literature and a series of focus groups conducted with carers. Thematic analysis of focus group data revealed specific difficulties and challenges experienced by carers in the early post-stroke aftermath, and helpful coping strategies commonly used. We also conducted a nominal group approach with stroke rehabilitation experts to further refine the intervention. The biopsychosocial intervention was designed to recognise and target the

For peer review only

difficulties commonly experienced by informal carers (identified through the focus groups and from the stroke literature). Additionally, helpful coping strategies used by the informal carers were used to further inform and adapt the content of the intervention. More detailed information about the development of the intervention will be provided in a further publication.

Intervention and Comparator

Participants are randomised as dyads. Participants will be randomised to either:

- **Control Group: usual care.** Carers randomised to the control group will receive the usual range of routine care and services available to them. They will not receive the biopsychosocial intervention.

- **Intervention Group: Biopsychosocial Intervention, plus usual care** Carers randomised to the intervention group will receive the biopsychosocial intervention, in addition to usual care. The stroke carers randomised to receive the intervention will receive a two-hour session once a week for six weeks. The time-point at which the intervention will start will be agreed with the carer, in conjunction with other carers likely to be part of that group. This will occur when the stroke survivor they care for has been discharged from hospital, up to one year post-stroke. We will aim to deliver the intervention to groups of approximately 5 people. However in the event that it is not possible to coordinate sufficient people, or where carers are unable to attend the group sessions, we will deliver the intervention on a one to one basis or in smaller groups. We will record this as part of our feasibility. The intervention will be delivered at a suitable venue, with sufficient space and access for carer group members. The intervention sessions will be facilitated by a research psychologist who has received accredited training in the principles of biopsychosocial theory as well as specific training from members of the research team responsible for the development of the intervention. Clinical supervision and debriefing sessions will be provided by an experienced community mental health nurse with significant Community Stroke Team experience, and/or a clinical psychologist. Each session will last approximately 2 hours and will include a 15-minute tea/coffee break which will allow participants to interact more informally with one another, and the session will conclude with a 15-minute relaxation exercise. We anticipate that in the

For peer review only - http://bmjopen.bmj.com/site/about/guidelines.xhtml

definitive trial, the intervention could be delivered by assistant psychologists with supervision from clinical psychologists.

The intervention programme is focused on adjustment to stroke, provision of psychoeducation and psychological support. The group programme is based on the principles of the biopsychosocial model. The sessions are designed to teach individuals to identify and use skills to reduce current and future distress, thus aiding coping and adjustment to the impact of stroke and their role as a carer. The sessions are also intended to increase awareness of the role of thoughts, emotions and behaviours and their influence on each other. By practising problem solving and stress-management strategies, it is hoped that carers will experience fewer difficulties with their mood in the future.

For each session there will be a presentation containing information about a topic and exercises to aid discussion. Sessions will be presented on Microsoft PowerPoint and all participants will receive either electronic or paper copies of the slides as appropriate, accompanied by the exercise and an in between session task. The topics will cover, for example, an introduction to stroke and caring, adjustment and mood, how to handle negative emotions and thoughts, dealing with problems, and a well-being relapse prevention plan (which provides a set of coping mechanisms to deal with individual triggers of the stress response in relation to the role as stroke carers to encourage and foster positive mental health). The content of these sessions was informed by the findings of earlier work described above. Relaxation exercises at the end of each session will allow participants to feel calm and relaxed before finishing their session, and also an effective tool for them to use outside of the session when experiencing high levels of anxiety or distress. Between session tasks will be set to encourage participants to practice exercises from the sessions in their own time. Participants who are identified to be experiencing significant issues that are outwith the scope of the intervention will be referred onto the appropriate specialist service, subject to their consent.

Outcomes

The main outcome for the study is to determine the feasibility of conducting a larger, powered study. This will be a composite of: whether the eligibility criteria are realistic; whether stroke survivors and carers are willing to be randomised; the study attrition rate; the feasibility and acceptability of delivering the intervention; the suitability and

For peer review only - http://bmjopen.bmj.com/site/about/guidelines.xhtml

sensitivity of outcome measures; the most suitable outcome measure for use in the main study.

The stroke carer outcomes to be assessed at six months post-randomisation, will be: anxiety and depression; health related quality of life; and carer strain. The outcome measures which will be used are: Hospital Anxiety and Depression Scale[35]; EuroQol EQ5D-5L[36], and the caregiver burden scale[37].

The stroke survivor outcomes to be assessed six months post-randomisation, will be: level of disability; ability to perform personal activities of daily living; level of anxiety and depression; health related quality of life. The outcomes measures which will be used are: Modified Rankin Scale[38]; Barthel Index[39]; Hospital Anxiety and Depression Scale[35]; EuroQol EQ5D-5L[36]. The timeline and proposed flow of participants through the study is shown in Figure 1.

Feedback interviews

Qualitative semi-structured interviews will be conducted with carers in both arms of the trial, within two weeks of their final outcome assessment. Up to 10 interviews will be completed with stroke carers in each arm. Our aim is to obtain feedback on all aspects of the study in addition to the intervention procedures, assessments, intervention (if received) and perceived outcomes. For those in the control group the interviews will provide confirmation of the nature of usual care received. Participants will be purposefully selected to include carers of stroke survivors with varying severity of stroke, age and gender. The interviews will be conducted by a researcher who had no involvement in the intervention delivery, thereby reducing social desirability response bias. The researcher conducting the interviews will become aware of the group allocation during the interview and so will not be masked to the intervention. These interviews will be audio recorded using a digital recorder, transcribed and analysed using a thematic analysis (following the procedure described by Braun and Clarke[40]). The interviews with participants will provide information feedback on their perception of progress over time and for those in the intervention group, the quality of the intervention provided, and as such will serve as a process measure. Insights from the qualitative data and analysis will serve to inform developments of the intervention programme in the future and to generate user-orientated proposals about areas for further investigations. This information will also inform us of any refinements to be made to the study procedures. An interview will also be conducted with the group facilitator after they have completed

For peer review only - http://bmjopen.bmj.com/site/about/guidelines.xhtml

all therapy. This interview will ask about the ease of delivery of the intervention according to the manual and any challenges.

Sample Size, Recruitment Strategy, Randomisation and Blinding

For a feasibility study, no formal sample size calculation is required. The aim is to recruit up to 40 dyads (20 in each arm of the trial) to test the randomisation process and the feasibility of the study processes of delivering the intervention. This target should allow us to collect sufficient information on the suitability and sensitivity of the outcome measures for use with this population and the standard deviations of the measures to inform a sample size calculation for a definitive trial. The median sample size for UK feasibility trials has been reported at 36[41] which is broadly consistent with the planned target.

The trial opened for recruitment on 1st November 2015 and will close on 31st July 2017 or when 40 dyads have been recruited (whichever is soonest). Participants will be enrolled into the study by a member of staff from the Clinical Research Network (CRN) or a member of the research team. The process for obtaining participant informed consent will be in accordance with the Research Ethics Committee (REC) guidance, and Good Clinical Practice (GCP) and any other regulatory requirements that might be introduced. Following a full explanation of the study, the participant will be required to provide informed written consent before they can participate. Where a consultee is required for a stroke survivor, the consultee shall provide a recommendation as to whether they consider the person would have agreed to take part in the study, had they still had capacity to state their own preference. They will sign the consultee declaration, should they believe that person would have wished to take part in the study.

Participants will be randomised at baseline following consent and completion of the baseline assessments. Randomisation to each group will be on a 1:1 basis, intervention:control. A simple randomisation procedure will be provided and overseen by the East Midlands Research Design Service. The group facilitator will be informed of group allocation as they will be providing the treatment. We will take every step to minimise allocation and outcome bias.

Trial participants will not be masked to group allocation because they will need to be informed as to whether they have been allocated to the intervention group receiving the biopsychosocial intervention, or the control group. The participants' names, trial identifier numbers and treatment allocation will be stored on a password protected

For peer review only - http://bmjopen.bmj.com/site/about/guidelines.xhtml

database held by the group facilitator. This database will be used to allow treatment allocations to be identified at the end of the study.

Baseline data will be collected and baseline assessments will be completed prior to randomisation. Baseline information will include:

1. Demographic details including age, gender, ethnicity and employment
2. Number and percentage of participants who meet eligibility criteria
3. Number of eligible participants who give consent
4. Levels of anxiety and depression (Hospital Anxiety and Depression Scale)
5. Quality of Life (EuroQol)
6. Carer strain (Carer Burden Scale).

In addition, we will collect the following information from the stroke survivor and/or their medical notes (with consent):

1. Stroke characteristics
2. Language and Cognitive Abilities (Montreal Cognitive Assessment)
3. Personal Activities of Daily Living (Barthel Index)
4. Stroke severity (National Institute of Health Stroke Scale)
5. Quality of Life (EuroQol)
6. Which service (if any) the stroke survivor is discharged to (e.g. ESD, intermediate care).

Follow-up assessment visits will be completed at six-months post-randomisation by a research assistant who is masked to allocation. To minimize the risk of unmasking, prior to each contact, the participant will be reminded that the researcher who is to conduct their follow-up assessment is masked. It is possible that participants may reveal their group allocation to the outcome assessors and any instances of this will be recorded by researchers as part of the assessment of feasibility; researchers will also be asked to make their 'best guess' as to the group allocation of the participants to determine whether masking was successful. Other members of the research team and investigators will not be masked to group allocation for the purpose of managing the trial and delivering the interventions. It will not be possible to mask participants.

Data Collection, Management and Analysis

Data will be collected on a paper case report form (CRF) and will subsequently be entered onto a secure password protected, purposely designed electronic database. Each participant will be assigned a trial identity code number, allocated at randomisation, for

For peer review only - http://bmjopen.bmj.com/site/about/guidelines.xhtml

use on CRFs other trial documents and the electronic database to ensure confidentiality. The documents and database will also use their initials and date of birth. CRFs will be treated as confidential documents and held securely in accordance with regulations.

When data collection is complete, a data quality check will be conducted in duplicate by two researchers and a 10% sample of the database will be checked against the original paper CRF. Steps will be taken to minimise missing data by personal contact throughout the study period from the investigator and every attempt will be made to locate participants for follow-up. Where participants are unavailable for follow-up, details of the attempts to contact them will be recorded. Outcome data will be collected in person, in the participant's home, by a research assistant to minimise the amount of missing data. For each outcome measure used where data is missing, an imputed average will be used for items where less than 10% of the overall measure is missing. Where more than 10% of a measure is missing, the entire measure will be coded as missing, unless the scoring criterion for that measure stipulates an alternative approach. We will not collect any further data for participants who withdraw from the study, but we will retain all data collected up until the point of withdrawal.

The following procedures will apply to data analysis:

Acceptability of the study design

Recruitment rates, proportion of carers screened who are eligible for enrolment and who provided consent, how easily carers can be identified, who met the criteria for the study, number of people who accepted intervention to take part in the RCT, number of individuals who attended the intervention/number of sessions they attended. The feedback interviews will provide further information regarding the acceptability of the intervention. Qualitative thematic analysis will provide an insight into carer perspectives of their experience of caring and what effect they think the intervention itself may have had (for the treatment group).

Feasibility of completing the intervention

Proportion of carers completing the assessment and interventions. Feedback interviews will also provide information about delivery of the intervention both from the perspective of the group facilitator and the experiences of the carers themselves.

Tolerability

For peer review only - http://bmjopen.bmj.com/site/about/guidelines.xhtml

Proportion of carers who withdraw or decline intervention. Record of interventions declined and why.

Integrity of the study protocol

By examining how many participants are able to complete the study, % of missing data, percentage of people who completed questionnaires, percentage of people who completed each outcome measures at 6 month follow up, calculation of the cost of running the study.

Outcome measures

Outcome measure data will be stored in a database and data will be analysed using the statistical package STATA. The proportion of missing items will be examined. The questionnaire data will be analysed to determine the distributions of scores. The analysis will use descriptive statistics and confidence intervals for the parameters we are estimating. The characteristics of stroke survivors and their carers will also be described using means, standard deviations and ranges for quantitative variables and counts and proportions for categorical variables. Data will be analysed on an 'intent to treat' basis. Any changes in the planned statistical methods will be documented in the report.

Ethics

Ethical approval for this study was provided by East Midlands – Nottingham 2 Research Ethics Committee (14/EMI/1264). Health Research Authority (HRA) and research and development approvals have been obtained as necessary.

DISSEMINATION

This study will provide the foundations and information needed to inform a further, appropriately powered study to investigate the effectiveness of the biopsychosocial intervention for stroke survivors and their carers. There are few interventional studies for stroke carers and this study is addressing a key area of concern for the stroke community. The findings will therefore be relevant to researchers, clinicians, commissioners, stroke survivors and carers. Additionally, there is an increasing focus on interventions which prevent or delay the need for other health and social care services; the findings will also be relevant to policymakers in this area.

We plan to disseminate our findings through presentations at national and international stroke and rehabilitation conferences, and we will submit findings for publication in a peer reviewed academic journal.

For peer review only - http://bmjopen.bmj.com/site/about/guidelines.xhtml

Trial Status

The trial is in recruitment phase. Recruitment commenced in November 2015; recruitment is due to close in July 2017. The trial is registered ISRCTN 15643456.

List of Abbreviations

BISC – Biopsychosocial Intervention for Stroke Carers

CRF – Case report form

CRN – Clinical Research Network

GCP – Good Clinical Practice

HRA – Health Research Authority

RCT – Randomised Controlled Trial

REC – Research Ethics Committee

Competing Interests

None declared.

Authors' Contributions

MW, ST, PW, RF and CC drafted the manuscript. MW, RF and CC conceived the study, MW is the principal investigator. LC contributed to the design of the study and delivers the intervention. MW, ST, EK and PB developed the intervention. All authors are members of the research team involved in the running of the study. All authors commented critically on the manuscript and read and approved the final manuscript.

Acknowledgements

We would like to thank the project steering group: Sheila Birchall, Charlotte Davies, Christopher Greensmith, Kate Hooban, Ben Jackson, Raj Mehta and Nikola Sprigg. We would also like to thank Joanna Fletcher-Smith, Miriam Golding-Day, Oliver Matias and Helen Taylor for their assistance with conducing the study.

Study Sponsor and Funder

The sponsor for this study is the University of Nottingham, Research and Graduate Services, King's Meadow Campus, Lenton Lane, Nottingham, NG7 2NR.

This protocol is independent research funded by the National Institute for Health Research (Research for Patient Benefit Programme, Biopsychosocial Intervention for Stroke Carers (BISC), PB-PG-0613-31064). The views expressed in this publication are

For peer review only - http://bmjopen.bmj.com/site/about/guidelines.xhtml

those of the authors and not necessarily those of the NHS, the National Institute for Health Research or the Department of Health.

For peer review only

For peer review only - http://bmjopen.bmj.com/site/about/guidelines.xhtml

References

1. Scottish Government Health Directorate. National Framework for Service Change in the NHS in Scotland. Self care, carers, volunteering and the voluntary sector: towards a more collaborative approach. Secondary National Framework for Service Change in the NHS in Scotland. Self care, carers, volunteering and the voluntary sector: towards a more collaborative approach 2005. http://www.sehd.scot.nhs.uk/NationalFramework/Documents/electivecare/Selfcare230505.pdf.

2. McCullagh E, Brigstocke G, Donaldson N, et al. Determinants of caregiving burden and quality of life in caregivers of stroke patients. Stroke (00392499) 2005;**36**(10):2181-86.

3. Han B, Haley WE. Family caregiving for patients with stroke. Review and analysis. Stroke 1999;**30**(7):1478-85.

4. Royal College of Physicians. Sentinel Stroke National Audit Programme (SSNAP) Clinical audit December 2016 - March 2017 Public Report. London: Royal College of Physicians, 2017.

5. Department of Health. Care and Support statutory guidance. Secondary Care and Support statutory guidance 2017. https://www.gov.uk/government/publications/care-act-statutory-guidance/care-and-support-statutory-guidance#fn:1.

6. Intercollegiate Stroke Working Party. National clinical guideline for stroke. Fifth ed. London: Royal College of Physicians, 2016.

7. National Institute for Health and Care Excellence. Stroke Rehabilitation in Adults. London: National Institute for Health and Care Excellence, 2013.

8. Greenwood N, Mackenzie A, Cloud GC, et al. Informal primary carers of stroke survivors living at home - Challenges, satisfactions and coping: A systematic review of qualitative studies. Disability and Rehabilitation 2009;**31**(5):337-51.

9. The Stroke Association. Feeling Overwhelmed: The emotional impact of stroke. Life After Stroke Compaign Report. London: The Stroke Association, 2013.

For peer review only - http://bmjopen.bmj.com/site/about/guidelines.xhtml

10. Greenwood N, Mackenzie A, Cloud GC, et al. Informal carers of stroke survivors - Factors influencing carers: A systematic review of quantitative studies. Disability and Rehabilitation 2008;**30**(18):1329-49.

11. Legg LA, Quinn TJ, Mahmood F, et al. Non-pharmacological interventions for caregivers of stroke survivors. Cochrane Database of Systematic Reviews 2011(10):CD008179.

12. Cheng HY, Chair SY, Chau JP. The effectiveness of psychosocial interventions for stroke family caregivers and stroke survivors: a systematic review and meta-analysis. Patient Educ Couns 2014;**95**(1):30-44.

13. Rubbens E, De Clerck L, Swinnen E. Effectiveness of Interventions to Decrease the Physical and Mental Burden and Strain of Informal Caregivers of Stroke Patients: A Systematic Review. Converging Clinical and Engineering Research on Neurorehabilitation II: Springer, 2017:299-303.

14. Greenwood N, Mackenzie A. Informal caring for stroke survivors: meta-ethnographic review of qualitative literature. Maturitas 2010;**66**(3):268-76.

15. Heslin M, Forster A, Healey A, et al. A systematic review of the economic evidence for interventions for family carers of stroke patients. Clin Rehabil 2016;**30**(2):119-33.

16. Anderson C, Mhurchu C, Rubenach S, et al. Hospital or home for stroke rehabilitation? Results of a randomised controlled trial. II: cost minimization analysis at 6 months. Stroke 2000;**31**:1032-37.

17. Lincoln NB, Francis VM, Lilley SA, et al. Evaluation of a stroke family support organiser: a randomized controlled trial. Stroke 2003;**34**(1):116-21.

18. Ellis G, Mant J, Langhorne P, et al. Stroke liaison workers for stroke patients and carers: an individual patient data meta-analysis. Cochrane Database of Systematic Reviews 2010(5):CD005066.

19. Greenwood N, Mackenzie A, Harris R, et al. Perceptions of the role of general practice and practical support measures for carers of stroke survivors: a qualitative study. BMC family practice 2011;**12**(1):57.

For peer review only

For peer review only - http://bmjopen.bmj.com/site/about/guidelines.xhtml

20. Smith LN, Lawrence M, Kerr SM, et al. Informal carers' experience of caring for stroke survivors. Journal of Advanced Nursing 2004;**46**(3):235-44.

21. Patel A, Knapp M, Evans A, et al. Training care givers of stroke patients: Economic evaluation. BMJ: British Medical Journal 2004;**328**(7448):1102.

22. Printz-Feddersen V. Group process effect on caregiver burden. Journal of Neuroscience Nursing 1990;**22**(3):164-68.

23. Forster A, Dickerson J, Young J, et al. A structured training programme for caregivers of inpatients after stroke (TRACS): a cluster randomised controlled trial and cost-effectiveness analysis. Lancet 2013;**382**(9910):2069-76.

24. Lovell K, Richards DA, Bower P. Improving access to primary mental health care: uncontrolled evaluation of a pilot self-help clinic. Br J Gen Pract 2003;**53**(487):133-35.

25. Kazdin AE, Blase SL. Rebooting psychotherapy research and practice to reduce the burden of mental illness. Perspectives on psychological science 2011;**6**(1):21-37.

26. Arksey H, Hirst M. Unpaid carers’ access to and use of primary care services. Primary Health Care Research and Development 2005;**6**(02):101-16.

27. Engel GL. The need for a new medical model: a challenge for biomedicine. Science 1977;**196**(4286):129-36.

28. Engel GL. The clinical application of the biopsychosocial model. Am J Psychiatry 1980;**137**(5):535-44.

29. Schotte CK, Van Den Bossche B, De Doncker D, et al. A biopsychosocial model as a guide for psychoeducation and treatment of depression. Depression and anxiety 2006;**23**(5):312-24.

30. Lundh U. Family carers 4: designing services to support family carers in Sweden. British Journal of Nursing 1999;**8**(12):787-90.

31. Mackenzie A, Perry L, Lockhart E, et al. Family carers of stroke survivors: needs, knowledge, satisfaction and competence in caring. Disability and Rehabilitation 2007;**29**(2):111-21.

For peer review only - http://bmjopen.bmj.com/site/about/guidelines.xhtml

32. Camak DJ. Addressing the burden of stroke caregivers: a literature review. Journal of Clinical Nursing 2015;**24**(17/18):2376-82.

33. van den Heuvel ET, de Witte LP, Schure LM, et al. Risk factors for burn-out in caregivers of stroke patients, and possibilities for intervention. Clinical Rehabilitation 2001;**15**(6):669-77.

34. Nilsen ES, Myrhaug HT, Johansen M, et al. Methods of consumer involvement in developing healthcare policy and research, clinical practice guidelines and patient information material. Cochrane Database Syst Rev 2006;**3**(3).

35. Zigmond AS, Snaith RP. The hospital anxiety and depression scale. Acta psychiatrica scandinavica 1983;**67**(6):361-70.

36. EuroQol Group. EuroQol – a new facility for the measurement of health-related quality of life. Health Policy 1990;**16**:199-208.

37. Zarit SH, Reever KE, Bach-Peterson J. Relatives of the Impaired Elderly: Correlates of Feelings of Burden. The Gerontologist 1980;**20**(6):649-55.

38. Bonita R, Beaglehole R. Modification of Rankin Scale: Recovery of motor function after stroke. Stroke 1988;**19**(12):1497-500.

39. Collin C, Wade D, Davies S, et al. The Barthel ADL Index: a reliability study. Disability & Rehabilitation 1988;**10**(2):61-63.

40. Braun V, Clarke V. Using thematic analysis in psychology. Qualitative Research in Psychology 2006;**3**(2):77-101.

41. Billingham SA, Whitehead AL, Julious SA. An audit of sample sizes for pilot and feasibility trials being undertaken in the United Kingdom registered in the United Kingdom Clinical Research Network database. BMC medical research methodology 2013;**13**(1):1.

Figure 1: FLOW of Participants through the study

For peer review only - http://bmjopen.bmj.com/site/about/guidelines.xhtml

For peer review only

For peer review only - http://bmjopen.bmj.com/site/about/guidelines.xhtml

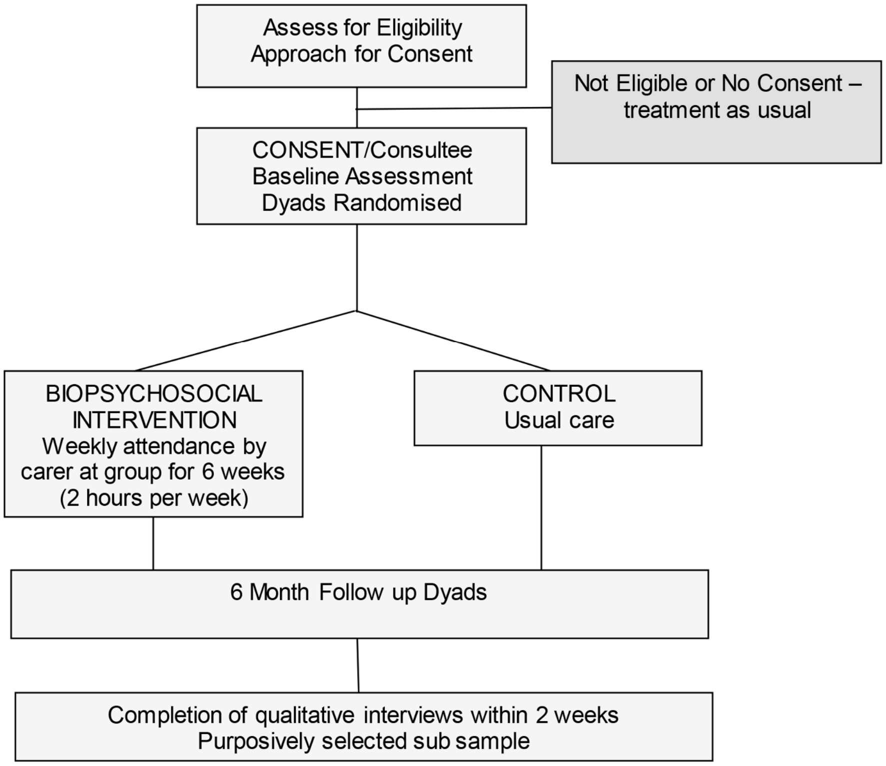

Figure 1: Flow of participants through the study

170x149mm (300 x 300 DPI)

12

STANDARD PROTOCOL ITEMS: RECOMMENDATIONS FOR INTERVENTIONAL TRIALS

SPIRIT 2013 Checklist: Recommended items to address in a clinical trial protocol and related documents*

Section/item	Item No	Description	Addressed on page number
**Administrative information**			
Title	1	Descriptive title identifying the study design, population, interventions, and, if applicable, trial acronym	___ 1___
Trial registration	2a	Trial identifier and registry name. If not yet registered, name of intended registry	_________1_____
	2b	All items from the World Health Organization Trial Registration Data Set	___Throughout__
Protocol version	3	Date and version identifier	_________1_____
Funding	4	Sources and types of financial, material, and other support	________16______
Roles and responsibilities	5a	Names, affiliations, and roles of protocol contributors	_____1 & 15____
	5b	Name and contact information for the trial sponsor	________16______
	5c	Role of study sponsor and funders, if any, in study design; collection, management, analysis, and interpretation of data; writing of the report; and the decision to submit the report for publication, including whether they will have ultimate authority over an4 - 6y of these activities	________16______
	5d	Composition, roles, and responsibilities of the coordinating centre, steering committee, endpoint adjudication committee, data management team, and other individuals or groups overseeing the trial, if applicable (see Item 21a for data monitoring committee)	_______N/A______

For peer review only - http://bmjopen.bmj.com/site/about/guidelines.xhtml

**Introduction**			
Background and rationale	6a	Description of research question and justification for undertaking the trial, including summary of relevant studies (published and unpublished) examining benefits and harms for each intervention	________4-6 ______
	6b	Explanation for choice of comparators	______8________
Objectives	7	Specific objectives or hypotheses	______6-7_______
Trial design	8	Description of trial design including type of trial (eg, parallel group, crossover, factorial, single group), allocation ratio, and framework (eg, superiority, equivalence, noninferiority, exploratory)	________7_______
**Methods: Participants, interventions, and outcomes**			
Study setting	9	Description of study settings (eg, community clinic, academic hospital) and list of countries where data will be collected. Reference to where list of study sites can be obtained	_________7______
Eligibility criteria	10	Inclusion and exclusion criteria for participants. If applicable, eligibility criteria for study centres and individuals who will perform the interventions (eg, surgeons, psychotherapists)	________7-8______
Interventions	11a	Interventions for each group with sufficient detail to allow replication, including how and when they will be administered	________8-10_____
	11b	Criteria for discontinuing or modifying allocated interventions for a given trial participant (eg, drug dose change in response to harms, participant request, or improving/worsening disease)	________9_______
	11c	Strategies to improve adherence to intervention protocols, and any procedures for monitoring adherence (eg, drug tablet return, laboratory tests)	_______10 &14____
	11d	Relevant concomitant care and interventions that are permitted or prohibited during the trial	_______8________
Outcomes	12	Primary, secondary, and other outcomes, including the specific measurement variable (eg, systolic blood pressure), analysis metric (eg, change from baseline, final value, time to event), method of aggregation (eg, median, proportion), and time point for each outcome. Explanation of the clinical relevance of chosen efficacy and harm outcomes is strongly recommended	________10_______
Participant timeline	13	Time schedule of enrolment, interventions (including any run-ins and washouts), assessments, and visits for participants. A schematic diagram is highly recommended (see Figure)	Figure 1 & 8-9

For peer review only - http://bmjopen.bmj.com/site/about/guidelines.xhtml

Sample size	14	Estimated number of participants needed to achieve study objectives and how it was determined, including clinical and statistical assumptions supporting any sample size calculations	______11_______
Recruitment	15	Strategies for achieving adequate participant enrolment to reach target sample size	_______11 -12___

Methods: Assignment of interventions (for controlled trials)

Allocation:

Sequence generation	16a	Method of generating the allocation sequence (eg, computer-generated random numbers), and list of any factors for stratification. To reduce predictability of a random sequence, details of any planned restriction (eg, blocking) should be provided in a separate document that is unavailable to those who enrol participants or assign interventions	______12_______
Allocation concealment mechanism	16b	Mechanism of implementing the allocation sequence (eg, central telephone; sequentially numbered, opaque, sealed envelopes), describing any steps to conceal the sequence until interventions are assigned	_______12______
Implementation	16c	Who will generate the allocation sequence, who will enrol participants, and who will assign participants to interventions	________12_____
Blinding (masking)	17a	Who will be blinded after assignment to interventions (eg, trial participants, care providers, outcome assessors, data analysts), and how	_______12-13 ___
	17b	If blinded, circumstances under which unblinding is permissible, and procedure for revealing a participant's allocated intervention during the trial	________N/A____

Methods: Data collection, management, and analysis

Data collection methods	18a	Plans for assessment and collection of outcome, baseline, and other trial data, including any related processes to promote data quality (eg, duplicate measurements, training of assessors) and a description of study instruments (eg, questionnaires, laboratory tests) along with their reliability and validity, if known. Reference to where data collection forms can be found, if not in the protocol	________13_____
	18b	Plans to promote participant retention and complete follow-up, including list of any outcome data to be collected for participants who discontinue or deviate from intervention protocols	_______13______

For peer review only - http://bmjopen.bmj.com/site/about/guidelines.xhtml

Data management	19	Plans for data entry, coding, security, and storage, including any related processes to promote data quality (eg, double data entry; range checks for data values). Reference to where details of data management procedures can be found, if not in the protocol	_______13_______
Statistical methods	20a	Statistical methods for analysing primary and secondary outcomes. Reference to where other details of the statistical analysis plan can be found, if not in the protocol	________13-14 ___
	20b	Methods for any additional analyses (eg, subgroup and adjusted analyses)	__________N/A___
	20c	Definition of analysis population relating to protocol non-adherence (eg, as randomised analysis), and any statistical methods to handle missing data (eg, multiple imputation)	________13______
**Methods: Monitoring**			
Data monitoring	21a	Composition of data monitoring committee (DMC); summary of its role and reporting structure; statement of whether it is independent from the sponsor and competing interests; and reference to where further details about its charter can be found, if not in the protocol. Alternatively, an explanation of why a DMC is not needed	__________N/A___
	21b	Description of any interim analyses and stopping guidelines, including who will have access to these interim results and make the final decision to terminate the trial	_______N/A_______
Harms	22	Plans for collecting, assessing, reporting, and managing solicited and spontaneously reported adverse events and other unintended effects of trial interventions or trial conduct	_______N/A_______
Auditing	23	Frequency and procedures for auditing trial conduct, if any, and whether the process will be independent from investigators and the sponsor	________N/A_____
**Ethics and dissemination**			
Research ethics approval	24	Plans for seeking research ethics committee/institutional review board (REC/IRB) approval	_________14____
Protocol amendments	25	Plans for communicating important protocol modifications (eg, changes to eligibility criteria, outcomes, analyses) to relevant parties (eg, investigators, REC/IRBs, trial participants, trial registries, journals, regulators)	_________N/A____

For peer review only - http://bmjopen.bmj.com/site/about/guidelines.xhtml

Consent or assent	26a	Who will obtain informed consent or assent from potential trial participants or authorised surrogates, and how (see Item 32)	________11______
	26b	Additional consent provisions for collection and use of participant data and biological specimens in ancillary studies, if applicable	_________N/A____
Confidentiality	27	How personal information about potential and enrolled participants will be collected, shared, and maintained in order to protect confidentiality before, during, and after the trial	________13______
Declaration of interests	28	Financial and other competing interests for principal investigators for the overall trial and each study site	_________15_____
Access to data	29	Statement of who will have access to the final trial dataset, and disclosure of contractual agreements that limit such access for investigators	________13______
Ancillary and post-trial care	30	Provisions, if any, for ancillary and post-trial care, and for compensation to those who suffer harm from trial participation	________N/A_____
Dissemination policy	31a	Plans for investigators and sponsor to communicate trial results to participants, healthcare professionals, the public, and other relevant groups (eg, via publication, reporting in results databases, or other data sharing arrangements), including any publication restrictions	________14-15___
	31b	Authorship eligibility guidelines and any intended use of professional writers	________15______
	31c	Plans, if any, for granting public access to the full protocol, participant-level dataset, and statistical code	________N/A_____
**Appendices**			
Informed consent materials	32	Model consent form and other related documentation given to participants and authorised surrogates	________N/A_____
Biological specimens	33	Plans for collection, laboratory evaluation, and storage of biological specimens for genetic or molecular analysis in the current trial and for future use in ancillary studies, if applicable	________N/A_____

*It is strongly recommended that this checklist be read in conjunction with the SPIRIT 2013 Explanation & Elaboration for important clarification on the items. Amendments to the protocol should be tracked and dated. The SPIRIT checklist is copyrighted by the SPIRIT Group under the Creative Commons "Attribution-NonCommercial-NoDerivs 3.0 Unported" license.

For peer review only - http://bmjopen.bmj.com/site/about/guidelines.xhtml

BMJ Open

Biopsychosocial Intervention for Stroke Carers (BISC): Protocol for a Feasibility Randomised Controlled Trial (RCT)

Journal:	*BMJ Open*
Manuscript ID	bmjopen-2017-018309.R2
Article Type:	Protocol
Date Submitted by the Author:	19-Sep-2017
Complete List of Authors:	Walker, Marion; Faculty of Medicine & Health Sciences, University of Nottingham<br>Thomas, Shirley<br>Whitehead, Phillip; University of Nottingham, Division of Rehabilitation and Ageing<br>Condon, Laura<br>Fisher, Rebecca; Univ Nottingham<br>Kontou, Eirini; University of Nottingham, Division of Rehabilitation & Ageing<br>Benford, Penny; University of Nottingham, c/o Division of Rehabilitation & Ageing<br>Cobley, Christine; Derbyshire Healthcare NHS Foundation Trust
**Primary Subject Heading**:	Health services research
Secondary Subject Heading:	Rehabilitation medicine, Neurology
Keywords:	Carers, Stroke < NEUROLOGY, Biospsychosocial model

SCHOLARONE™ Manuscripts

only

For peer review only - http://bmjopen.bmj.com/site/about/guidelines.xhtml

Biopsychosocial Intervention for Stroke Carers (BISC): Protocol for a Feasibility Randomised Controlled Trial (RCT)

Marion F Walker*
Division of Rehabilitation and Ageing, University of Nottingham
B Floor, The Medical School, Queens Medical Centre, Nottingham, NG7 2UH
marion.walker@nottingham.ac.uk

Shirley A Thomas
Division of Rehabilitation and Ageing, University of Nottingham
B Floor, The Medical School, Queens Medical Centre, Nottingham, NG7 2UH
shirley.thomas@nottingham.ac.uk

Phillip J Whitehead
Division of Rehabilitation and Ageing, University of Nottingham
B Floor, The Medical School, Queens Medical Centre, Nottingham, NG7 2UH
phillip.j.whitehead@nottingham.ac.uk

Laura Condon
Division of Rehabilitation and Ageing, University of Nottingham
B Floor, The Medical School, Queens Medical Centre, Nottingham, NG7 2UH
laura.condon@nottingham.ac.uk

Rebecca Fisher
Division of Rehabilitation and Ageing, University of Nottingham
B Floor, The Medical School, Queens Medical Centre, Nottingham, NG7 2UH
rebecca.fisher@nottingham.ac.uk

Eirini Kontou
Division of Rehabilitation and Ageing, University of Nottingham
B Floor, The Medical School, Queens Medical Centre, Nottingham, NG7 2UH
eirini.kontou@nottingham.ac.uk

Penny Benford
Division of Rehabilitation and Ageing, University of Nottingham
B Floor, The Medical School, Queens Medical Centre, Nottingham, NG7 2UH
penny.benford@nottingham.ac.uk

Christine Cobley
Department of Clinical Psychology, Derbyshire Healthcare NHS Foundation Trust, St Andrews House, 201 London Road, Derby, DE1 2TZ.
Christine.cobley@derbyshcft.nhs.uk

*Corresponding Author

Word Count (main text): 4267

Protocol Version 5.0 17.10.16

For peer review only - http://bmjopen.bmj.com/site/about/guidelines.xhtml

Abstract

Introduction

Reducing length of hospital stay for stroke survivors often creates a shift in the responsibility of care towards informal carers. Adjustment to the caregiving process is experienced by many carers as overwhelming, complex and demanding, and can have a detrimental impact on mental and physical health and wellbeing. National policy guidelines recommend that carers' needs are considered and addressed; despite this, few interventions have been developed and empirically evaluated. We developed a biopsychosocial intervention in collaboration with carers of stroke survivors. Our aim is to determine whether the intervention can be delivered in a group setting and evaluated using a randomised controlled trial (RCT).

Methods and Analysis

Feasibility randomised controlled trial (RCT) and nested qualitative interview study. We aim to recruit up to 40 stroke carers within one year of the stroke onset. Carers are randomised to usual care or usual care plus biopsychosocial intervention. Each intervention group will consist of five stroke carers. The intervention will focus on: psychoeducation, psychological adjustment to stroke, strategies for reducing unwanted negative thoughts and emotions, and problem solving strategies. The main outcome is the feasibility of conducting an RCT. Carer outcomes at six months include: anxiety and depression, quality of life, and carer strain. Data is also collected from stroke survivors at baseline and six months including: level of disability, anxiety and depression, and quality of life.

Ethics and Dissemination

Favourable ethical opinion was provided by East Midlands – Nottingham2 Research Ethics Committee (14/EMI/1264). This study will determine whether delivery of the biopsychosocial intervention is feasible and acceptable to stroke carers within a group format. It will also determine whether it is feasible to evaluate the effects of the biopsychosocial intervention in an RCT. We will disseminate our findings through peer-reviewed publications and presentations at national and international conferences.

Trial Registration: ISRCTN15643456

For peer review only - http://bmjopen.bmj.com/site/about/guidelines.xhtml

Strengths and limitations of this study

- Enhancing the wellbeing of carers is a national priority. However, few interventions for carers have been developed or evaluated.
- This is a pragmatic trial conducted in a real world setting. The intervention content is based on the findings from the literature, developmental work with carers of stroke survivors and stroke rehabilitation experts.
- This feasibility study will be conducted in a single site only.
- The intervention focusses only on the initial stages of carer support (up to one year post-stroke onset). Significant problems may develop for carers at later stages which need to be identified and referred for more intensive/specialist support.

For peer review only

For peer review only - http://bmjopen.bmj.com/site/about/guidelines.xhtml

INTRODUCTION

Background and Rationale

A carer has been defined as "a person of any age who provides unpaid help and support to a relative, friend or neighbour who cannot manage to live independently without the carer's help due to frailty, illness, disability or addiction"[1]. Carers play a vital role in the early rehabilitation process and long-term management of the stroke survivor[2]. Carers deal with a range of care needs and demands including mobility, self-care, communication difficulties as well as cognitive impairment, mood and personality changes in the stroke survivor[3].

The latest figures from the Sentinel Stroke National Audit in England, Wales and Northern Ireland show that the median length of inpatient stay is between 7 and 8 days; however, just under one third of stroke survivors who were discharged requiring help with daily activities received assistance from informal carers[4]. This demonstrates that increasingly shorter hospital stays coincide with an earlier transfer of care to informal carers in the community. The Care Act 2014 has placed a statutory responsibility on local authorities in England to consider the wellbeing of carers as being of equal importance to the wellbeing of the people they care for[5]. The importance of providing support and intervention to carers has also been emphasised in national stroke guidelines[6 7]. Consequently, it is becoming more urgent to develop appropriate and effective interventions to meet the specific needs of carers of stroke survivors.

A growing number of people are unexpectedly finding themselves in the caregiving role. Although it can be a positive and rewarding role[8], the increased demands associated with informal caring can place carers at elevated risk of poorer mental and physical health, accompanied by reduced opportunity for paid employment and social interaction and activity[3]. An estimate of the psychosocial impact that might be associated with stroke care, drawn from a survey of carers[9], shows that carers may experience: anxiety (79%), frustration (84%), sleeping disturbances (60%), depression (56%), and stress (57%). Deterioration in the health and wellbeing of the carer has important implications on the outcomes of stroke survivors including: poorer rehabilitation outcomes; reduced quality of life; heightened levels of depression; greater risk of mortality; poorer treatment adherence; and increased likelihood of being placed into institutional care, which has important cost implications for the NHS, social care services, the stroke survivor and their family.

For peer review only - http://bmjopen.bmj.com/site/about/guidelines.xhtml

An increased focus on the needs of stroke carers has led to a spate of recent systematic reviews including quantitative and interventional studies[10-13], qualitative research[8 14] and economic evidence[15]. However, the results are equivocal and limited. Thus, there is no clear and robust evidence regarding the most effective and cost effective interventions for stroke carers. This lack of evidence is not due to the lack of research in this field[11]; interventions directed at both stroke survivors and carers forms the largest body of research and has predominantly focused upon examining new models of service delivery[16], such as care-giver training, the stroke family support worker[17 18], and multidisciplinary hospital and community stroke teams. These interventions however have predominantly focused upon the stroke survivors needs rather than the carers needs[19], and thus the needs of carers have largely been neglected. The few interventions to date that have been developed specifically for carers include education and information[20], skills training[21] and social support[22]. Such interventions however have produced inconclusive findings, arguably because such interventions are failing to address and meet the specific needs of carers. Forster et al[23] evaluated a structured caregiver training programme delivered in hospital by multidisciplinary teams from stroke units. There was no difference between the intervention and usual care and they concluded that the immediate period after stroke might not be the best time to deliver such a programme.

Given the high prevalence of psychological morbidity within the stroke carer population, there is likely to be a high demand for psychologically informed interventions targeted at informal carers of stroke survivors beyond the initial period of hospitalisation. Although evidence-based treatments for psychological difficulties exist, the associated costs and expenses of service delivery are high, with demand for treatment exceeding service capacity, resulting in long waiting lists[24] and limited access[25]. There are other barriers experienced by carers wishing to pursue and access mental health services[26]. These barriers include a lack of attention by health professionals of the difficulties associated with the caregiving role, and that general practitioners are often more likely to offer practical rather than psychological support. Together these reasons make it increasingly difficult for informal carers to access evidence-based psychologically informed interventions.

The biopsychosocial model of health and illness, as proposed by Engel[27], suggests that psychobiological vulnerability is influenced by an interaction of biological (physical health), psychological (thoughts, emotions and behaviours) and social (relationships

For peer review only - http://bmjopen.bmj.com/site/about/guidelines.xhtml

and roles) factors. The model emphasises the need for interventions to focus on both symptom reduction and on relapse prevention[28]. Psychological models such as cognitive-behavioural and interpersonal therapy have been deemed too fragmented and reductionist[29], given that they do not integrate the biological and psychological factors, as well as social, environmental and stress factors that are known to interfere with psychological functioning.

There have been movements towards the use of biopsychosocial interventions for the treatment of psychological difficulties amongst the general population. However, evidence suggests that significant adaptations to such interventions are required prior to application to different clinical populations. Indeed, mental health services for carers have been criticised for not being tailored to address the unique and specific difficulties experienced by stroke carers[30 31]. Such difficulties can include having to manage the physical and cognitive impairment and behavioural difficulties the stroke survivor may be presenting with[32 33]. There is growing recognition of the importance of understanding carer's experiences when dealing with health resources and healthcare policy[34]. A systematic review of psychosocial interventions for stroke carers concluded that more randomised controlled trials of psychoeducation programmes are needed[12].

Considering this recommendation in the context of the wider literature on stroke carers, we developed a new biopsychosocial intervention specifically targeted at informal carers of stroke survivors. The intervention was developed collaboratively with stroke carers and designed to be delivered in a group format to offer participants the opportunity to meet and interact with people and listen to how others have coped. Delivering the intervention in a group format is also likely to be more time and cost efficient, which would be important given the current demand for psychological therapies.

This study is examining the feasibility of conducting a randomised controlled trial (RCT) to examine the effectiveness and acceptability of this group biopsychosocial intervention for stroke carers in the first year post-stroke.

Research Aim and Objectives

The ultimate aim of this study is to evaluate whether a biopsychosocial intervention can improve psychological outcomes in carers of stroke survivors (in the one year post-stroke period). However, we are not able to complete a definitive, powered trial until we have collected further information to inform the design of such a study. The purpose of

For peer review only - http://bmjopen.bmj.com/site/about/guidelines.xhtml

this feasibility trial is to explore whether the biopsychosocial intervention for carers of stroke survivors is feasible, acceptable and to estimate the parameters for conducting a fully powered trial.

Primary Objective

The primary objective of this feasibility trial is to evaluate whether it is feasible to deliver a biopsychosocial intervention to carers of stroke survivors as part of a randomised controlled trial (RCT).

Secondary Objectives

This feasibility RCT will test the integrity of the study protocol, such as the methods of data collection, randomisation procedures and the masking of independent assessors. This feasibility study will answer the necessary questions to inform a definitive multi-centre trial which include:

- Can we identify participants willing to be randomised?
- Can we deliver the intervention as planned?
- Is the intervention acceptable to participants?
- Can we retain participants in the study?
- What are the most relevant outcome measures?
- What is the consent rate?

METHODS AND ANALYSIS

Study Design and Setting

This is a single centre feasibility RCT with nested qualitative interview study. The RCT is a parallel group, two arm trial with a 1:1 allocation ratio biopsychosocial intervention: usual care control.

Participants

Participants are carers of people who have had a stroke (stroke survivors). Our definition of a carer is a family member or friend who is/will be providing support for a stroke survivor who would not be able to manage without their help due to their condition. Carers will be recruited along with stroke survivors from stroke units at a University Hospital, community stroke services, and third sector stroke clubs and support groups. However, only the carer will receive the intervention; we will recruit the stroke survivor because we also aim to collect baseline and follow-up data from them.

The inclusion criteria are as follows:

For peer review only

Stroke carers:

- Aged 18 or over
- Carer of a person with a confirmed diagnosis of stroke within one year of stroke onset
- Capacity to provide informed consent
- Willing to attend a 6 week group intervention programme

Stroke survivors:

- Aged 18 or over
- Confirmed diagnosis of stroke
- Within one year of stroke onset
- Capacity to provide informed consent or consultee opinion that the person would wish to participate

The exclusion criteria are as follows:

Stroke carers:

- Unable to speak English
- Engaged in other research involving biopsychosocial/psychological interventions
- People with visual (blindness) or auditory (deafness) impairments that would preclude them from participating in the therapy sessions.

Stroke survivors:

- Unable to speak English
- People engaged in other research involving biopsychosocial/psychological interventions

Intervention Development

The intervention was developed based on the biopsychosocial model of health and illness[27] with the aim to address biological, psychological and social factors and symptom reduction and relapse prevention. The content was informed through a review of the literature and a series of focus groups conducted with carers. Thematic analysis of focus group data revealed specific difficulties and challenges experienced by carers in the early post-stroke aftermath, and helpful coping strategies commonly used. We also conducted a nominal group approach with stroke rehabilitation experts to further refine the intervention. The biopsychosocial intervention was designed to recognise and target the

For peer review only - http://bmjopen.bmj.com/site/about/guidelines.xhtml

difficulties commonly experienced by informal carers (identified through the focus groups and from the stroke literature). Additionally, helpful coping strategies used by the informal carers were used to further inform and adapt the content of the intervention. More detailed information about the development of the intervention will be provided in a further publication.

Intervention and Comparator

Participants are randomised as dyads. Participants will be randomised to either:

- **Control Group: usual care.** Carers randomised to the control group will receive the usual range of routine care and services available to them. They will not receive the biopsychosocial intervention.

- **Intervention Group: Biopsychosocial Intervention, plus usual care**
  Carers randomised to the intervention group will receive the biopsychosocial intervention, in addition to usual care. The stroke carers randomised to receive the intervention will receive a two-hour session once a week for six weeks. The time-point at which the intervention will start will be agreed with the carer, in conjunction with other carers likely to be part of that group. This will occur when the stroke survivor they care for has been discharged from hospital, up to one year post-stroke. We will aim to deliver the intervention to groups of approximately 5 people. However in the event that it is not possible to coordinate sufficient people, or where carers are unable to attend the group sessions, we will deliver the intervention on a one to one basis or in smaller groups. We will record this as part of our feasibility. The intervention will be delivered at a suitable venue, with sufficient space and access for carer group members. The intervention sessions will be facilitated by a research psychologist who has received training in the principles of biopsychosocial theory as well as specific training from members of the research team responsible for the development of the intervention. Clinical supervision and debriefing sessions will be provided by an experienced community mental health nurse with significant Community Stroke Team experience, and/or a clinical psychologist. Each session will last approximately 2 hours and will include a 15-minute tea/coffee break which will allow participants to interact more informally with one another, and the session will conclude with a 15-minute relaxation exercise. We anticipate that in the definitive trial, the

For peer review only - http://bmjopen.bmj.com/site/about/guidelines.xhtml

intervention could be delivered by assistant psychologists with supervision from clinical psychologists.

The intervention programme is focused on adjustment to stroke, provision of psychoeducation and psychological support. The group programme is based on the principles of the biopsychosocial model. The sessions are designed to teach individuals to identify and use skills to reduce current and future distress, thus aiding coping and adjustment to the impact of stroke and their role as a carer. The sessions are also intended to increase awareness of the role of thoughts, emotions and behaviours and their influence on each other. By practising problem solving and stress-management strategies, it is hoped that carers will experience fewer difficulties with their mood in the future.

For each session there will be a presentation containing information about a topic and exercises to aid discussion. Sessions will be presented on Microsoft PowerPoint and all participants will receive either electronic or paper copies of the slides as appropriate, accompanied by the exercise and an in between session task. The topics will cover, for example, an introduction to stroke and caring, adjustment and mood, how to handle negative emotions and thoughts, dealing with problems, and a well-being relapse prevention plan (which provides a set of coping mechanisms to deal with individual triggers of the stress response in relation to the role as stroke carers to encourage and foster positive mental health). The content of these sessions was informed by the findings of earlier work described above. Relaxation exercises at the end of each session will allow participants to feel calm and relaxed before finishing their session, and also an effective tool for them to use outside of the session when experiencing high levels of anxiety or distress. Between session tasks will be set to encourage participants to practice exercises from the sessions in their own time. Participants who are identified to be experiencing significant issues that are outwith the scope of the intervention will be referred onto the appropriate specialist service, subject to their consent.

Outcomes

The main outcome for the study is to determine the feasibility of conducting a larger, powered study. This will be a composite of: whether the eligibility criteria are realistic; whether stroke survivors and carers are willing to be randomised; the study attrition rate; the feasibility and acceptability of delivering the intervention; the suitability and

For peer review only - http://bmjopen.bmj.com/site/about/guidelines.xhtml

sensitivity of outcome measures; the most suitable outcome measure for use in the main study.

The stroke carer outcomes to be assessed at six months post-randomisation, will be: anxiety and depression; health related quality of life; and carer strain. The outcome measures which will be used are: Hospital Anxiety and Depression Scale[35]; EuroQol EQ5D-5L[36], and the caregiver burden scale[37].

The stroke survivor outcomes to be assessed six months post-randomisation, will be: level of disability; ability to perform personal activities of daily living; level of anxiety and depression; health related quality of life. The outcomes measures which will be used are: Modified Rankin Scale[38]; Barthel Index[39]; Hospital Anxiety and Depression Scale[35]; EuroQol EQ5D-5L[36]. The timeline and proposed flow of participants through the study is shown in Figure 1.

Feedback interviews

Qualitative semi-structured interviews will be conducted with carers in both arms of the trial, within two weeks of their final outcome assessment. Up to 10 interviews will be completed with stroke carers in each arm. Our aim is to obtain feedback on all aspects of the study in addition to the intervention procedures, assessments, intervention (if received) and perceived outcomes. For those in the control group the interviews will provide confirmation of the nature of usual care received. Participants will be purposefully selected to include carers of stroke survivors with varying severity of stroke, age and gender. The interviews will be conducted by a researcher who had no involvement in the intervention delivery, thereby reducing social desirability response bias. The researcher conducting the interviews will become aware of the group allocation during the interview and so will not be masked to the intervention. These interviews will be audio recorded using a digital recorder, transcribed and analysed using a thematic analysis (following the procedure described by Braun and Clarke[40]). The interviews with participants will provide information feedback on their perception of progress over time and for those in the intervention group, the quality of the intervention provided, and as such will serve as a process measure. Insights from the qualitative data and analysis will serve to inform developments of the intervention programme in the future and to generate user-orientated proposals about areas for further investigations. This information will also inform us of any refinements to be made to the study procedures. An interview will also be conducted with the group facilitator after they have completed

For peer review only

For peer review only - http://bmjopen.bmj.com/site/about/guidelines.xhtml

all therapy. This interview will ask about the ease of delivery of the intervention according to the manual and any challenges.

Sample Size, Recruitment Strategy, Randomisation and Blinding

For a feasibility study, no formal sample size calculation is required. The aim is to recruit up to 40 dyads (20 in each arm of the trial) to test the randomisation process and the feasibility of the study processes of delivering the intervention. This target should allow us to collect sufficient information on the suitability and sensitivity of the outcome measures for use with this population and the standard deviations of the measures to inform a sample size calculation for a definitive trial. The median sample size for UK feasibility trials has been reported at 36[41] which is broadly consistent with the planned target.

The trial opened for recruitment on 1st November 2015 and will close on 31st July 2017 or when 40 dyads have been recruited (whichever is soonest). Participants will be enrolled into the study by a member of staff from the Clinical Research Network (CRN) or a member of the research team. The process for obtaining participant informed consent will be in accordance with the Research Ethics Committee (REC) guidance, and Good Clinical Practice (GCP) and any other regulatory requirements that might be introduced. Following a full explanation of the study, the participant will be required to provide informed written consent before they can participate. Where a consultee is required for a stroke survivor, the consultee shall provide a recommendation as to whether they consider the person would have agreed to take part in the study, had they still had capacity to state their own preference. They will sign the consultee declaration, should they believe that person would have wished to take part in the study.

Participants will be randomised at baseline following consent and completion of the baseline assessments. Randomisation to each group will be on a 1:1 basis, intervention:control. A simple randomisation procedure will be provided and overseen by the East Midlands Research Design Service. The group facilitator will be informed of group allocation as they will be providing the treatment. We will take every step to minimise allocation and outcome bias.

Trial participants will not be masked to group allocation because they will need to be informed as to whether they have been allocated to the intervention group receiving the biopsychosocial intervention, or the control group. The participants' names, trial identifier numbers and treatment allocation will be stored on a password protected

For peer review only - http://bmjopen.bmj.com/site/about/guidelines.xhtml

database held by the group facilitator. This database will be used to allow treatment allocations to be identified at the end of the study.

Baseline data will be collected and baseline assessments will be completed prior to randomisation. Baseline information will include:

1. Demographic details including age, gender, ethnicity and employment
2. Number and percentage of participants who meet eligibility criteria
3. Number of eligible participants who give consent
4. Levels of anxiety and depression (Hospital Anxiety and Depression Scale)
5. Quality of Life (EuroQol)
6. Carer strain (Carer Burden Scale).

In addition, we will collect the following information from the stroke survivor and/or their medical notes (with consent):

1. Stroke characteristics
2. Language and Cognitive Abilities (Montreal Cognitive Assessment)
3. Personal Activities of Daily Living (Barthel Index)
4. Stroke severity (National Institute of Health Stroke Scale)
5. Quality of Life (EuroQol)
6. Which service (if any) the stroke survivor is discharged to (e.g. ESD, intermediate care).

Follow-up assessment visits will be completed at six-months post-randomisation by a research assistant who is masked to allocation. To minimize the risk of unmasking, prior to each contact, the participant will be reminded that the researcher who is to conduct their follow-up assessment is masked. It is possible that participants may reveal their group allocation to the outcome assessors and any instances of this will be recorded by researchers as part of the assessment of feasibility; researchers will also be asked to make their 'best guess' as to the group allocation of the participants to determine whether masking was successful. Other members of the research team and investigators will not be masked to group allocation for the purpose of managing the trial and delivering the interventions. It will not be possible to mask participants.

Data Collection, Management and Analysis

Data will be collected on a paper case report form (CRF) and will subsequently be entered onto a secure password protected, purposely designed electronic database. Each participant will be assigned a trial identity code number, allocated at randomisation, for

For peer review only - http://bmjopen.bmj.com/site/about/guidelines.xhtml

use on CRFs other trial documents and the electronic database to ensure confidentiality. The documents and database will also use their initials and date of birth. CRFs will be treated as confidential documents and held securely in accordance with regulations.

When data collection is complete, a data quality check will be conducted in duplicate by two researchers and a 10% sample of the database will be checked against the original paper CRF. Steps will be taken to minimise missing data by personal contact throughout the study period from the investigator and every attempt will be made to locate participants for follow-up. Where participants are unavailable for follow-up, details of the attempts to contact them will be recorded. Outcome data will be collected in person, in the participant's home, by a research assistant to minimise the amount of missing data. For each outcome measure used where data is missing, an imputed average will be used for items where less than 10% of the overall measure is missing. Where more than 10% of a measure is missing, the entire measure will be coded as missing, unless the scoring criterion for that measure stipulates an alternative approach. We will not collect any further data for participants who withdraw from the study, but we will retain all data collected up until the point of withdrawal.

The following procedures will apply to data analysis:

Acceptability of the study design

Descriptive statistics will be presented for the following feasibility outcomes: recruitment rates, proportion of carers screened who are eligible for enrolment and who provided consent, how easily carers can be identified, who met the criteria for the study, number of people who accepted intervention to take part in the RCT, number of individuals who attended the intervention/number of sessions they attended. The feedback interviews will provide further information regarding the acceptability of the intervention. Qualitative thematic analysis will provide an insight into carer perspectives of their experience of caring and what effect they think the intervention itself may have had (for the treatment group).

Feasibility of completing the intervention

Proportion of carers completing the assessment and interventions. Feedback interviews will also provide information about delivery of the intervention both from the perspective of the group facilitator and the experiences of the carers themselves.

Tolerability

For peer review only - http://bmjopen.bmj.com/site/about/guidelines.xhtml

Proportion of carers who withdraw or decline intervention. Record of interventions declined and why.

Integrity of the study protocol

By examining how many participants are able to complete the study, % of missing data, percentage of people who completed questionnaires, percentage of people who completed each outcome measures at 6 month follow up, calculation of the cost of running the study.

Outcome measures

Outcome measure data will be stored in a database and data will be analysed using the statistical package STATA. The proportion of missing items will be examined. The questionnaire data will be analysed to determine the distributions of scores. The analysis will use descriptive statistics and confidence intervals for the parameters we are estimating. The characteristics of stroke survivors and their carers will also be described using means, standard deviations and ranges for quantitative variables and counts and proportions for categorical variables. Data will be analysed on an 'intent to treat' basis. Any changes in the planned statistical methods will be documented in the report.

Ethics

Ethical approval for this study was provided by East Midlands – Nottingham 2 Research Ethics Committee (14/EMI/1264). Health Research Authority (HRA) and research and development approvals have been obtained as necessary.

DISSEMINATION

This study will provide the foundations and information needed to inform a further, appropriately powered study to investigate the effectiveness of the biopsychosocial intervention for stroke survivors and their carers. There are few interventional studies for stroke carers and this study is addressing a key area of concern for the stroke community. The findings will therefore be relevant to researchers, clinicians, commissioners, stroke survivors and carers. Additionally, there is an increasing focus on interventions which prevent or delay the need for other health and social care services; the findings will also be relevant to policymakers in this area.

We plan to disseminate our findings through presentations at national and international stroke and rehabilitation conferences, and we will submit findings for publication in a peer reviewed academic journal.

For peer review only

For peer review only - http://bmjopen.bmj.com/site/about/guidelines.xhtml

Trial Status

The trial is in recruitment phase. Recruitment commenced in November 2015; recruitment is due to close in July 2017. The trial is registered ISRCTN 15643456.

List of Abbreviations

BISC – Biopsychosocial Intervention for Stroke Carers

CRF – Case report form

CRN – Clinical Research Network

GCP – Good Clinical Practice

HRA – Health Research Authority

RCT – Randomised Controlled Trial

REC – Research Ethics Committee

Competing Interests

None declared.

Authors' Contributions

MW, ST, PW, RF and CC drafted the manuscript. MW, RF and CC conceived the study, MW is the principal investigator. LC contributed to the design of the study and delivers the intervention. MW, ST, EK and PB developed the intervention. All authors are members of the research team involved in the running of the study. All authors commented critically on the manuscript and read and approved the final manuscript.

Acknowledgements

We would like to thank the project steering group: Sheila Birchall, Charlotte Davies, Christopher Greensmith, Kate Hooban, Ben Jackson, Raj Mehta and Nikola Sprigg. We would also like to thank Joanna Fletcher-Smith, Miriam Golding-Day, Oliver Matias and Helen Taylor for their assistance with conducing the study.

Study Sponsor and Funder

The sponsor for this study is the University of Nottingham, Research and Graduate Services, King's Meadow Campus, Lenton Lane, Nottingham, NG7 2NR.

This protocol is independent research funded by the National Institute for Health Research (Research for Patient Benefit Programme, Biopsychosocial Intervention for Stroke Carers (BISC), PB-PG-0613-31064). The views expressed in this publication are

For peer review only - http://bmjopen.bmj.com/site/about/guidelines.xhtml

those of the authors and not necessarily those of the NHS, the National Institute for Health Research or the Department of Health.

For peer review only

For peer review only - http://bmjopen.bmj.com/site/about/guidelines.xhtml

References

1. Scottish Government Health Directorate. National Framework for Service Change in the NHS in Scotland. Self care, carers, volunteering and the voluntary sector: towards a more collaborative approach. Secondary National Framework for Service Change in the NHS in Scotland. Self care, carers, volunteering and the voluntary sector: towards a more collaborative approach 2005. http://www.sehd.scot.nhs.uk/NationalFramework/Documents/electivecare/Selfcare230505.pdf.

2. McCullagh E, Brigstocke G, Donaldson N, et al. Determinants of caregiving burden and quality of life in caregivers of stroke patients. Stroke (00392499) 2005;**36**(10):2181-86.

3. Han B, Haley WE. Family caregiving for patients with stroke. Review and analysis. Stroke 1999;**30**(7):1478-85.

4. Royal College of Physicians. Sentinel Stroke National Audit Programme (SSNAP) Clinical audit December 2016 - March 2017 Public Report. London: Royal College of Physicians, 2017.

5. Department of Health. Care and Support statutory guidance. Secondary Care and Support statutory guidance 2017. https://www.gov.uk/government/publications/care-act-statutory-guidance/care-and-support-statutory-guidance#fn:1.

6. Intercollegiate Stroke Working Party. National clinical guideline for stroke. Fifth ed. London: Royal College of Physicians, 2016.

7. National Institute for Health and Care Excellence. Stroke Rehabilitation in Adults. London: National Institute for Health and Care Excellence, 2013.

8. Greenwood N, Mackenzie A, Cloud GC, et al. Informal primary carers of stroke survivors living at home - Challenges, satisfactions and coping: A systematic review of qualitative studies. Disability and Rehabilitation 2009;**31**(5):337-51.

9. The Stroke Association. Feeling Overwhelmed: The emotional impact of stroke. Life After Stroke Compaign Report. London: The Stroke Association, 2013.

For peer review only

For peer review only - http://bmjopen.bmj.com/site/about/guidelines.xhtml

10. Greenwood N, Mackenzie A, Cloud GC, et al. Informal carers of stroke survivors - Factors influencing carers: A systematic review of quantitative studies. Disability and Rehabilitation 2008;**30**(18):1329-49.

11. Legg LA, Quinn TJ, Mahmood F, et al. Non-pharmacological interventions for caregivers of stroke survivors. Cochrane Database of Systematic Reviews 2011(10):CD008179.

12. Cheng HY, Chair SY, Chau JP. The effectiveness of psychosocial interventions for stroke family caregivers and stroke survivors: a systematic review and meta-analysis. Patient Educ Couns 2014;**95**(1):30-44.

13. Rubbens E, De Clerck L, Swinnen E. Effectiveness of Interventions to Decrease the Physical and Mental Burden and Strain of Informal Caregivers of Stroke Patients: A Systematic Review. Converging Clinical and Engineering Research on Neurorehabilitation II: Springer, 2017:299-303.

14. Greenwood N, Mackenzie A. Informal caring for stroke survivors: meta-ethnographic review of qualitative literature. Maturitas 2010;**66**(3):268-76.

15. Heslin M, Forster A, Healey A, et al. A systematic review of the economic evidence for interventions for family carers of stroke patients. Clin Rehabil 2016;**30**(2):119-33.

16. Anderson C, Mhurchu C, Rubenach S, et al. Hospital or home for stroke rehabilitation? Results of a randomised controlled trial. II: cost minimization analysis at 6 months. Stroke 2000;**31**:1032-37.

17. Lincoln NB, Francis VM, Lilley SA, et al. Evaluation of a stroke family support organiser: a randomized controlled trial. Stroke 2003;**34**(1):116-21.

18. Ellis G, Mant J, Langhorne P, et al. Stroke liaison workers for stroke patients and carers: an individual patient data meta-analysis. Cochrane Database of Systematic Reviews 2010(5):CD005066.

19. Greenwood N, Mackenzie A, Harris R, et al. Perceptions of the role of general practice and practical support measures for carers of stroke survivors: a qualitative study. BMC family practice 2011;**12**(1):57.

For peer review only

For peer review only - http://bmjopen.bmj.com/site/about/guidelines.xhtml

20. Smith LN, Lawrence M, Kerr SM, et al. Informal carers' experience of caring for stroke survivors. Journal of Advanced Nursing 2004;**46**(3):235-44.

21. Patel A, Knapp M, Evans A, et al. Training care givers of stroke patients: Economic evaluation. BMJ: British Medical Journal 2004;**328**(7448):1102.

22. Printz-Feddersen V. Group process effect on caregiver burden. Journal of Neuroscience Nursing 1990;**22**(3):164-68.

23. Forster A, Dickerson J, Young J, et al. A structured training programme for caregivers of inpatients after stroke (TRACS): a cluster randomised controlled trial and cost-effectiveness analysis. Lancet 2013;**382**(9910):2069-76.

24. Lovell K, Richards DA, Bower P. Improving access to primary mental health care: uncontrolled evaluation of a pilot self-help clinic. Br J Gen Pract 2003;**53**(487):133-35.

25. Kazdin AE, Blase SL. Rebooting psychotherapy research and practice to reduce the burden of mental illness. Perspectives on psychological science 2011;**6**(1):21-37.

26. Arksey H, Hirst M. Unpaid carers' access to and use of primary care services. Primary Health Care Research and Development 2005;**6**(02):101-16.

27. Engel GL. The need for a new medical model: a challenge for biomedicine. Science 1977;**196**(4286):129-36.

28. Engel GL. The clinical application of the biopsychosocial model. Am J Psychiatry 1980;**137**(5):535-44.

29. Schotte CK, Van Den Bossche B, De Doncker D, et al. A biopsychosocial model as a guide for psychoeducation and treatment of depression. Depression and anxiety 2006;**23**(5):312-24.

30. Lundh U. Family carers 4: designing services to support family carers in Sweden. British Journal of Nursing 1999;**8**(12):787-90.

31. Mackenzie A, Perry L, Lockhart E, et al. Family carers of stroke survivors: needs, knowledge, satisfaction and competence in caring. Disability and Rehabilitation 2007;**29**(2):111-21.

For peer review only - http://bmjopen.bmj.com/site/about/guidelines.xhtml

32. Camak DJ. Addressing the burden of stroke caregivers: a literature review. Journal of Clinical Nursing 2015;**24**(17/18):2376-82.

33. van den Heuvel ET, de Witte LP, Schure LM, et al. Risk factors for burn-out in caregivers of stroke patients, and possibilities for intervention. Clinical Rehabilitation 2001;**15**(6):669-77.

34. Nilsen ES, Myrhaug HT, Johansen M, et al. Methods of consumer involvement in developing healthcare policy and research, clinical practice guidelines and patient information material. Cochrane Database Syst Rev 2006;**3**(3).

35. Zigmond AS, Snaith RP. The hospital anxiety and depression scale. Acta psychiatrica scandinavica 1983;**67**(6):361-70.

36. EuroQol Group. EuroQol – a new facility for the measurement of health-related quality of life. Health Policy 1990;**16**:199-208.

37. Zarit SH, Reever KE, Bach-Peterson J. Relatives of the Impaired Elderly: Correlates of Feelings of Burden. The Gerontologist 1980;**20**(6):649-55.

38. Bonita R, Beaglehole R. Modification of Rankin Scale: Recovery of motor function after stroke. Stroke 1988;**19**(12):1497-500.

39. Collin C, Wade D, Davies S, et al. The Barthel ADL Index: a reliability study. Disability & Rehabilitation 1988;**10**(2):61-63.

40. Braun V, Clarke V. Using thematic analysis in psychology. Qualitative Research in Psychology 2006;**3**(2):77-101.

41. Billingham SA, Whitehead AL, Julious SA. An audit of sample sizes for pilot and feasibility trials being undertaken in the United Kingdom registered in the United Kingdom Clinical Research Network database. BMC medical research methodology 2013;**13**(1):1.

For peer review only - http://bmjopen.bmj.com/site/about/guidelines.xhtml

Figure 1: FLOW of Participants through the study

For peer review only

For peer review only - http://bmjopen.bmj.com/site/about/guidelines.xhtml

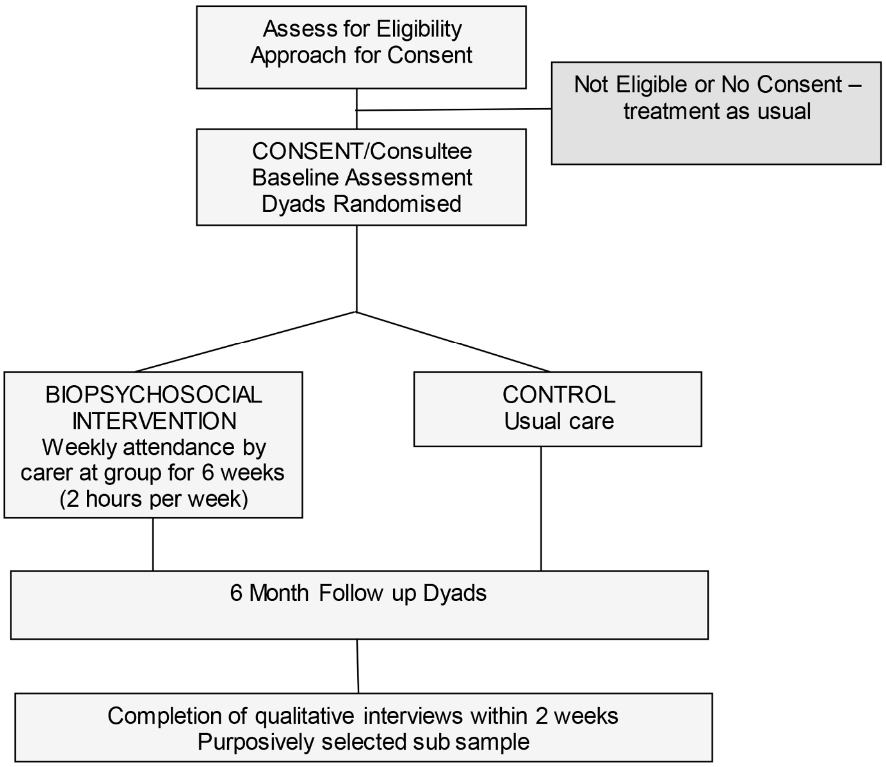

Figure 1: Flow of participants through the study

170x149mm (300 x 300 DPI)

only

12

SPIRIT

STANDARD PROTOCOL ITEMS: RECOMMENDATIONS FOR INTERVENTIONAL TRIALS

SPIRIT 2013 Checklist: Recommended items to address in a clinical trial protocol and related documents*

Section/item	Item No	Description	Addressed on page number
**Administrative information**			
Title	1	Descriptive title identifying the study design, population, interventions, and, if applicable, trial acronym	___ 1___
Trial registration	2a	Trial identifier and registry name. If not yet registered, name of intended registry	_________1_____
	2b	All items from the World Health Organization Trial Registration Data Set	___Throughout__
Protocol version	3	Date and version identifier	_________1_____
Funding	4	Sources and types of financial, material, and other support	________16______
Roles and responsibilities	5a	Names, affiliations, and roles of protocol contributors	_____1 & 15____
	5b	Name and contact information for the trial sponsor	________16______
	5c	Role of study sponsor and funders, if any, in study design; collection, management, analysis, and interpretation of data; writing of the report; and the decision to submit the report for publication, including whether they will have ultimate authority over an4 - 6y of these activities	________16______
	5d	Composition, roles, and responsibilities of the coordinating centre, steering committee, endpoint adjudication committee, data management team, and other individuals or groups overseeing the trial, if applicable (see Item 21a for data monitoring committee)	_______N/A______

For peer review only

For peer review only - http://bmjopen.bmj.com/site/about/guidelines.xhtml

Introduction

Background and rationale	6a	Description of research question and justification for undertaking the trial, including summary of relevant studies (published and unpublished) examining benefits and harms for each intervention	________4-6 ______
	6b	Explanation for choice of comparators	______8________
Objectives	7	Specific objectives or hypotheses	______6-7_______
Trial design	8	Description of trial design including type of trial (eg, parallel group, crossover, factorial, single group), allocation ratio, and framework (eg, superiority, equivalence, noninferiority, exploratory)	________7_______

Methods: Participants, interventions, and outcomes

Study setting	9	Description of study settings (eg, community clinic, academic hospital) and list of countries where data will be collected. Reference to where list of study sites can be obtained	_________7______
Eligibility criteria	10	Inclusion and exclusion criteria for participants. If applicable, eligibility criteria for study centres and individuals who will perform the interventions (eg, surgeons, psychotherapists)	________7-8______
Interventions	11a	Interventions for each group with sufficient detail to allow replication, including how and when they will be administered	________8-10_____
	11b	Criteria for discontinuing or modifying allocated interventions for a given trial participant (eg, drug dose change in response to harms, participant request, or improving/worsening disease)	________9_______
	11c	Strategies to improve adherence to intervention protocols, and any procedures for monitoring adherence (eg, drug tablet return, laboratory tests)	_______10 &14____
	11d	Relevant concomitant care and interventions that are permitted or prohibited during the trial	_______8________
Outcomes	12	Primary, secondary, and other outcomes, including the specific measurement variable (eg, systolic blood pressure), analysis metric (eg, change from baseline, final value, time to event), method of aggregation (eg, median, proportion), and time point for each outcome. Explanation of the clinical relevance of chosen efficacy and harm outcomes is strongly recommended	________10_______
Participant timeline	13	Time schedule of enrolment, interventions (including any run-ins and washouts), assessments, and visits for participants. A schematic diagram is highly recommended (see Figure)	Figure 1 & 8-9

Sample size	14	Estimated number of participants needed to achieve study objectives and how it was determined, including clinical and statistical assumptions supporting any sample size calculations	______11_______
Recruitment	15	Strategies for achieving adequate participant enrolment to reach target sample size	_______11 -12___
**Methods: Assignment of interventions (for controlled trials)**			
Allocation:			
Sequence generation	16a	Method of generating the allocation sequence (eg, computer-generated random numbers), and list of any factors for stratification. To reduce predictability of a random sequence, details of any planned restriction (eg, blocking) should be provided in a separate document that is unavailable to those who enrol participants or assign interventions	______12_______
Allocation concealment mechanism	16b	Mechanism of implementing the allocation sequence (eg, central telephone; sequentially numbered, opaque, sealed envelopes), describing any steps to conceal the sequence until interventions are assigned	_______12______
Implementation	16c	Who will generate the allocation sequence, who will enrol participants, and who will assign participants to interventions	________12_____
Blinding (masking)	17a	Who will be blinded after assignment to interventions (eg, trial participants, care providers, outcome assessors, data analysts), and how	_______12-13 ___
	17b	If blinded, circumstances under which unblinding is permissible, and procedure for revealing a participant's allocated intervention during the trial	________N/A____
**Methods: Data collection, management, and analysis**			
Data collection methods	18a	Plans for assessment and collection of outcome, baseline, and other trial data, including any related processes to promote data quality (eg, duplicate measurements, training of assessors) and a description of study instruments (eg, questionnaires, laboratory tests) along with their reliability and validity, if known. Reference to where data collection forms can be found, if not in the protocol	________13_____
	18b	Plans to promote participant retention and complete follow-up, including list of any outcome data to be collected for participants who discontinue or deviate from intervention protocols	_______13______

For peer review only - http://bmjopen.bmj.com/site/about/guidelines.xhtml

Data management	19	Plans for data entry, coding, security, and storage, including any related processes to promote data quality (eg, double data entry; range checks for data values). Reference to where details of data management procedures can be found, if not in the protocol	_______13_______
Statistical methods	20a	Statistical methods for analysing primary and secondary outcomes. Reference to where other details of the statistical analysis plan can be found, if not in the protocol	________13-14 ___
	20b	Methods for any additional analyses (eg, subgroup and adjusted analyses)	__________N/A___
	20c	Definition of analysis population relating to protocol non-adherence (eg, as randomised analysis), and any statistical methods to handle missing data (eg, multiple imputation)	________13______
**Methods: Monitoring**			
Data monitoring	21a	Composition of data monitoring committee (DMC); summary of its role and reporting structure; statement of whether it is independent from the sponsor and competing interests; and reference to where further details about its charter can be found, if not in the protocol. Alternatively, an explanation of why a DMC is not needed	__________N/A___
	21b	Description of any interim analyses and stopping guidelines, including who will have access to these interim results and make the final decision to terminate the trial	_______N/A_______
Harms	22	Plans for collecting, assessing, reporting, and managing solicited and spontaneously reported adverse events and other unintended effects of trial interventions or trial conduct	_______N/A_______
Auditing	23	Frequency and procedures for auditing trial conduct, if any, and whether the process will be independent from investigators and the sponsor	________N/A______
**Ethics and dissemination**			
Research ethics approval	24	Plans for seeking research ethics committee/institutional review board (REC/IRB) approval	_________14____
Protocol amendments	25	Plans for communicating important protocol modifications (eg, changes to eligibility criteria, outcomes, analyses) to relevant parties (eg, investigators, REC/IRBs, trial participants, trial registries, journals, regulators)	_________N/A____

For peer review only - http://bmjopen.bmj.com/site/about/guidelines.xhtml

Consent or assent	26a	Who will obtain informed consent or assent from potential trial participants or authorised surrogates, and how (see Item 32)	________11______
	26b	Additional consent provisions for collection and use of participant data and biological specimens in ancillary studies, if applicable	_________N/A____
Confidentiality	27	How personal information about potential and enrolled participants will be collected, shared, and maintained in order to protect confidentiality before, during, and after the trial	________13______
Declaration of interests	28	Financial and other competing interests for principal investigators for the overall trial and each study site	_________15_____
Access to data	29	Statement of who will have access to the final trial dataset, and disclosure of contractual agreements that limit such access for investigators	________13______
Ancillary and post-trial care	30	Provisions, if any, for ancillary and post-trial care, and for compensation to those who suffer harm from trial participation	________N/A_____
Dissemination policy	31a	Plans for investigators and sponsor to communicate trial results to participants, healthcare professionals, the public, and other relevant groups (eg, via publication, reporting in results databases, or other data sharing arrangements), including any publication restrictions	________14-15___
	31b	Authorship eligibility guidelines and any intended use of professional writers	________15______
	31c	Plans, if any, for granting public access to the full protocol, participant-level dataset, and statistical code	________N/A_____
**Appendices**			
Informed consent materials	32	Model consent form and other related documentation given to participants and authorised surrogates	________N/A_____
Biological specimens	33	Plans for collection, laboratory evaluation, and storage of biological specimens for genetic or molecular analysis in the current trial and for future use in ancillary studies, if applicable	________N/A_____

*It is strongly recommended that this checklist be read in conjunction with the SPIRIT 2013 Explanation & Elaboration for important clarification on the items. Amendments to the protocol should be tracked and dated. The SPIRIT checklist is copyrighted by the SPIRIT Group under the Creative Commons "Attribution-NonCommercial-NoDerivs 3.0 Unported" license.

For peer review only - http://bmjopen.bmj.com/site/about/guidelines.xhtml
